# Distinct Epigenetic Effects of Tobacco Smoking in Whole Blood and among Leukocyte Subtypes

**DOI:** 10.1371/journal.pone.0166486

**Published:** 2016-12-09

**Authors:** Dan Su, Xuting Wang, Michelle R. Campbell, Devin K. Porter, Gary S. Pittman, Brian D. Bennett, Ma Wan, Neal A. Englert, Christopher L. Crowl, Ryan N. Gimple, Kelly N. Adamski, Zhiqing Huang, Susan K. Murphy, Douglas A. Bell

**Affiliations:** 1 Environmental Genomics Group, Genome Integrity and Structural Biology Laboratory, National Institute of Environmental Health Sciences, National Institutes of Health, Research Triangle Park, NC, 27709, United States of America; 2 Integrated Bioinformatics, National Institute of Environmental Health Sciences, National Institutes of Health, Research Triangle Park, NC, 27709, United States of America; 3 Duke University School of Medicine, Durham, NC, 27708, United States of America; New York University School of Medicine, UNITED STATES

## Abstract

Tobacco smoke exposure dramatically alters DNA methylation in blood cells and may mediate smoking-associated complex diseases through effects on immune cell function. However, knowledge of smoking effects in specific leukocyte subtypes is limited. To better characterize smoking–associated methylation changes in whole blood and leukocyte subtypes, we used Illumina 450K arrays and Reduced Representation Bisulfite Sequencing (RRBS) to assess genome-wide DNA methylation. Differential methylation analysis in whole blood DNA from 172 smokers and 81 nonsmokers revealed 738 CpGs, including 616 previously unreported CpGs, genome-wide significantly associated with current smoking (*p* <1.2x10^-7^, Bonferroni correction). Several CpGs (*MTSS1*, *NKX6-2*, *BTG2*) were associated with smoking duration among heavy smokers (>22 cigarettes/day, n = 86) which might relate to long-term heavy-smoking pathology. In purified leukocyte subtypes from an independent group of 20 smokers and 14 nonsmokers we further examined methylation and gene expression for selected genes among CD14+ monocytes, CD15+ granulocytes, CD19+ B cells, and CD2+ T cells. In 10 smokers and 10 nonsmokers we used RRBS to fine map differential methylation in CD4+ T cells, CD8+ T cells, CD14+, CD15+, CD19+, and CD56+ natural killer cells. Distinct cell-type differences in smoking-associated methylation and gene expression were identified. *AHRR* (cg05575921), *ALPPL2* (cg21566642), *GFI1* (cg09935388), *IER3* (cg06126421) and *F2RL3* (cg03636183) showed a distinct pattern of significant smoking-associated methylation differences across cell types: granulocytes> monocytes>> B cells. In contrast *GPR15* (cg19859270) was highly significant in T and B cells and *ITGAL* (cg09099830) significant only in T cells. Numerous other CpGs displayed distinctive cell-type responses to tobacco smoke exposure that were not apparent in whole blood DNA. Assessing the overlap between these CpG sites and differential methylated regions (DMRs) with RRBS in 6 cell types, we confirmed cell-type specificity in the context of DMRs. We identified new CpGs associated with current smoking, pack-years, duration, and revealed unique profiles of smoking-associated DNA methylation and gene expression among immune cell types, providing potential clues to hematopoietic lineage-specific effects in disease etiology.

## Introduction

Tobacco smoke has pro-inflammatory and immunosuppressive effects [1] and is a major environmental risk factor for adverse health outcomes including lung cancer, chronic obstructive pulmonary disease, cardiovascular disease, arthritis, and type 2 diabetes. At the cellular level, tobacco smoke exposure induces DNA damage [2] and influences mutation frequency [3–5], and recent findings indicate smoking drives acquired differences in 5-methyl cytosine levels in blood cells and other tissues [6–8]. Despite a diversity of study designs used and populations examined, numerous recent epigenome-wide association studies (EWAS) [8–17] have identified repeatable, smoking-associated DNA methylation differences in whole blood DNA at CpGs located in or near genes including *AHRR*, *F2RL3*, *ALPPL2*, *IER3*, and *GPR15*. Studies of *in utero* tobacco exposure [18] and of recent new smokers [19] suggest *AHRR* methylation is altered even from short-term, low-dose exposure. Another study focusing on adult smokers suggested that epigenetic changes in inflammation genes might be related to long-term smoking [10] and the present work explores if heavy, long-term smoking produces epigenetic effects not seen in light smokers.

Blood leukocytes display characteristic transcription, chromatin, and DNA methylation patterns associated with their immune functions [20]. Smoking is known to affect immune cell function [1] and composition [21], and epigenetic studies utilizing whole blood may be detecting changes in activated immune cell subsets [22,23] or in specific leukocyte cell proportions. It is well recognized that these changes may confound or affect interpretation of results and useful algorithmic approaches for adjustment for cell type changes have been developed [22,24–30]. As Birney et al [27] recently point out, detailed epigenetic studies assessing exposure and disease effects on DNA methylation in specific cell types are needed in order to understand the meaning of EWAS results.

We hypothesized that cell-lineage dependent methylation responses to smoking were likely given the well-characterized differences in chromatin state, capacity for immunological activation, cell lifetime and other parameters that differ among leukocyte cell types. However, to date there is still no clear experimental evidence examining if exposure-driven DNA methylation effects may differ by leukocyte subtype. Smoking-related methylation changes in particular cell types could indicate different sensitivities to exposure and differing modes of action among cell lineages as well as potential functional effects that are important to cell-type specific disease etiology or to early detection of disease. With the exception of *AHRR* (cg05575921), in which methylation changes were observed to be significantly altered in smoker-derived lymphoblastoid cells and lung macrophages[7], or CD14+ monocytes and CD4+ T cells [31], most established smoking-associated CpG sites such as *F2RL3* (cg03636183), *ALPPL2* (cg21566642), *IER3* (cg06126421) and *GPR15* (cg19859270) have not been evaluated in multiple cell types. We expect that a more complete characterization of the relationship between differentially methylated regions (DMRs), chromatin context and transcription will help in elucidating the meaning of observed effects in whole blood and may reveal functional effects on immune cell subtypes.

We measured blood DNA methylation in 253 healthy subjects, including 86 heavy smokers with ≥ 28 pack-years, 86 smokers with < 28 pack-years and 81 nonsmokers, and identified CpG sites associated with current and cumulative smoking status and analyzed for effects of smoking duration among long-term, heavy current smokers. To explore the relationship between smoking-associated, cell type-specific methylation effects and leukocyte composition we conducted analysis of DNA methylation at candidate smoking-related loci (*AHRR*, *F2RL3*, *GPR15*, *ALPPL2*, *IER3*, *GFI1*, *MYO1G*, *ITGAL*) in purified CD14+ monocytes, CD15+ granulocytes, CD19+ B cells, and CD2+ T cells isolated from a separate population of 34 individuals. We further validated several of these as differentially methylated regions (DMRs) using Reduced Representation Bisulfite Sequencing (RRBS) to fine map them in CD14+, CD15+, CD19+, CD4+ T cells, CD8+ T cells, and CD56+ natural killer cells. This study extends the list of smoking-associated DNA methylation sites observed in whole blood DNA, particularly in heavy smokers. Importantly for a number of specific CpGs, we identify distinct patterns of response to smoking across leukocyte cell types and demonstrate how response in individual cell types impacts the effects observed in whole blood. We also compare smoking-associated methylation changes with transcriptional effects on nearby genes and histone modifications to identify lineage-dependent responses. The results identify the hematopoietic lineages responsible for important tobacco smoke-associated methylation changes detected in whole blood.

## Results

### Differential methylation between smokers and nonsmokers in DNA from whole blood

**Fig 1:**
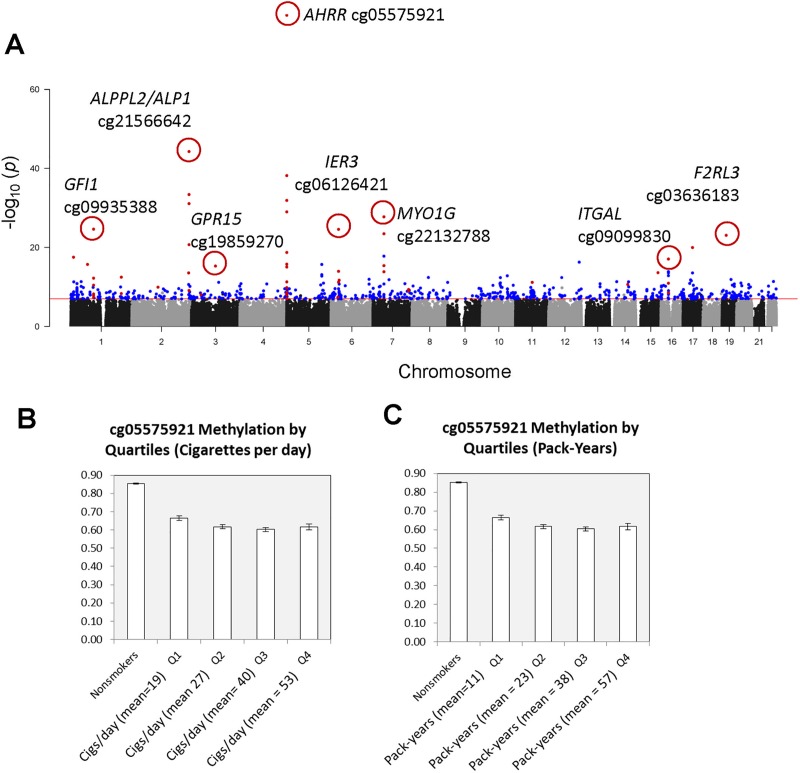
CpG methylation in whole blood DNA of heavy smokers. (A) Manhattan plot of p-value associations between smoking and CpG methylation with genome-wide significance level (red line, p = 1.5x10^-7^). CpGs in genes that were also examined in individual cell types are labeled. Blue points represent 1st time reported smoking CpGs. (B) AHRR cg05575921 methylation level by quartiles of self-reported cigarettes per day. (C) AHRR cg05575921 methylation level by quartiles of cumulative smoking in pack-year.

We assessed DNA methylation levels in whole blood DNA samples from current (SM, n = 172) and never smokers (NS, n = 81) and applied a multivariate robust linear regression model [32] to adjust for race, age, gender, and cell-type count (Tables 1 and 2) [22]. We observed 738 CpG sites associated with any level or duration of current smoking at genome-wide significance (p<1.2x10^-7^) (Fig 1, S1 Table). The most significant smoking-related DNA methylation site was in *AHRR* (cg05575921) with an adjusted p-value of 1.76x10^-79^ and 19 additional probes located in several *AHRR* DMRs reached genome-wide significance. One hundred twenty-two of 738 significant probes were reported previously (see S1 Table). The remaining 616 significant CpG sites (Fig 1A, colored blue) are first time reported. A number of newly reported genes displayed multiple highly significant CpGs including *NCOR2* (cg13015710, p = 5.50x10^-17^ and 2 others listed in S3 Table), *HMHB1* (cg02228160, p = 2.09x10^-16^), *RARA/RARA-AS1* (cg08446900, p = 2.89x10^-14^), *LMO7* (cg10581837, p = 1.59x10^-11^ and six others), and *SPOCK2* (cg00661320, p = 4.49x10^-13^) (see S3 Table). Other notable significant, smoking-associated CpGs included several within the *HOX* gene clusters, *RUNX3* (seven CpGs), and cg13940444 in *RARG* (p = 4.35x10^-13^).

**Table 1:** Characteristics of whole blood DNA methylation study participants.

Characteristic	Non-smokers (n = 81)	Smokers (n = 172)
Male/Female	44/37	127/45
Caucasian/African-American	63/18	126/46
Age, average, (SD, range)	31.6 (8.2, 19–53)	35.8 (8.8, 20–63)
Cigarettes per day, average (SD, range)	0	35.1 (14.1, 10–90)
Years smoked, average (SD, range)	0	17.3 (8.6, 2–42)
Pack-years, average (SD, range)	0	32.5(23.3, 1.8–114)
CD14+ Monocytes^a^	8.0%	7.3%
CD15+ Granulocytes^a^	50.9%	53.9%
CD19+ B Cells^a^	8.2%	8.1%
CD2+ T Cells^a^	35.2%	33.2%

Abbreviations: SD: standard deviation

^a^Cell-type distribution estimated by model (Houseman et al. 2012).

**Table 2:** Characteristics of cell-lineage DNA methylation participants.

^a^Characteristic	Non-smokers (n = 14)	Smokers (n = 20)	CBC Change with Smoking
Race, Caucasian/African–American	6/8	9/11	
Age, average (SD)	45.4 (8.4)	44.8 (9.3)	
Cotinine (ng/mL), average (SD)	3.8 (6.7)	227.4 (142.8)	
Cigarettes per day, average (SD)	0	28.3 (9.8)	
Years Smoked, average (SD)	0	29.6 (10.4)	
Pack-years, average (SD)	0	31.5 (21.3)	
Complete Blood Count (CBC)			
Lymphocytes (total), average (SD)	36.6% (7.1)	30.3% (11.6)	-6/3%
Monocytes, average (SD)	6.3% (3.8)	5.6% (2.3)	-0.7%
Neutrophils average (SD)	54.1 (7.3)	60.2% (11.9)	+6.1%
Eosinophils, average (SD)	2.3% (1.8)	3.2% (3.2)	+1.0%
Basophils, average (SD)	0.3% (0.5)	0.2% (0.4)	-0.1%
Large Unstained Cells, average (SD)	0.6% (1.1)	0.6% (0.9)	-

^a^Female participants in the cell fractionation study (n = 34) for analysis of cell-lineage specific DNA methylation.

### Genes with smoking-associated CpGs are enriched for hematological cancer and cardiovascular disease pathways

In these predominantly heavy smokers, a large number of CpGs are first-time reported. We employed GREAT (Genomic Regions Enrichment of Annotations Tool; Stanford University) to analyze the functional significance of differentially methylated CpGs in possible cis-regulatory regions. The 738 CpG sites were mapped onto genes within 100-kb and were highly enriched in disease ontology categories including hematologic cancer (enrichment score 3.09, binomial p = 1.28x10^-24^), cardiovascular system disease (2.43, p = 1.66x10^-23^), hematopoietic system disease (2.85, p = 7.61x10^-23^) and nervous system cancer (2.64, p = 1.83x10^-18^) (see Table 3; S2 and S3 Tables).

**Table 3:** GREAT Analysis of 738 Smoking Associated CpGs.

Category	Ontology	p-value	FDR	Enrichment
Disease Ontology	hematologic cancer	1.28x10^-24^	5.72x10^-22^	3.09
Disease Ontology	hematopoietic system disease	7.61x10^-23^	2.43x10^-20^	2.85
Disease Ontology	nervous system cancer	1.83x10^-18^	3.41x10^-16^	2.64
Disease Ontology	cardiovascular system disease	1.66x10^-23^	6.17x10^-21^	2.43

### Differential methylation associated with cumulative smoking

Examining pack-years (packs/day x years of smoking) on DNA methylation in all smokers resulted in substantially fewer significant smoking-associated CpGs. Of 738 sites associated with current smoking status, only 38 were genome-wide significantly associated with smoking pack-years after adjusting for age, race, gender, and cell-type count (see S1 Table, column M). Most have been observed in multiple studies including cg05575921 in *AHRR* (p = 1.68x10^-26^), cg21566642 near *ALPPL2* (p = 2.70x10^-19^), cg18146737 in *GFI1* (p = 2.44x10^-18^) and additional CpGs in *GPR15*, *AHRR*, *ALPPL2*, *F2RL3*, and *IER3*. There were three new smoking-associated sites, cg18826637 (*GTDC1*), cg09560590 (*HMHB1*), and cg08446900 *(RARA/RARA-AS1*) significantly associated (see S3 Table) with cumulative smoking (pack-years). Most CpGs showed decreased methylation relative to increasing pack-years, however, the effect of cumulative pack-year dose or years of smoking was modest. Fig 1B and 1C show current and cumulative dose/response patterns by quartile for *AHRR* cg05575921 and this trend was observed for many smoking-associated CpGs. This result suggests that any level of smoking appears to impact methylation level of *AHRR* but higher current or cumulative levels of smoking exposure produces very little added effect. Because long-term, heavy smokers are at the highest risk of smoking-associated disease we explored if the CpGs associated with long duration of heavy smoking might be different than the top CpG markers of any level of smoking. We carried out a stratified analysis (above and below median level of current smoking, <22 cigarettes/day versus ≥22 cigarettes/day) for years of smoking and ranked CpGs by the difference in rank between each stratum (S4 Table). These CpGs were genome-wide significant for years of smoking among heavy smokers, but nominally significant in lighter smokers. Rank changed most for cg24838345 within *MTSS1* (Metastasis Suppressor 1), cg15653173 located within *SOX1* and *LINC00403*, and cg11068946 in *NKX6-2*.

### Lineage-specific methylation differences for smoking-associated CpGs

**Fig 2:**
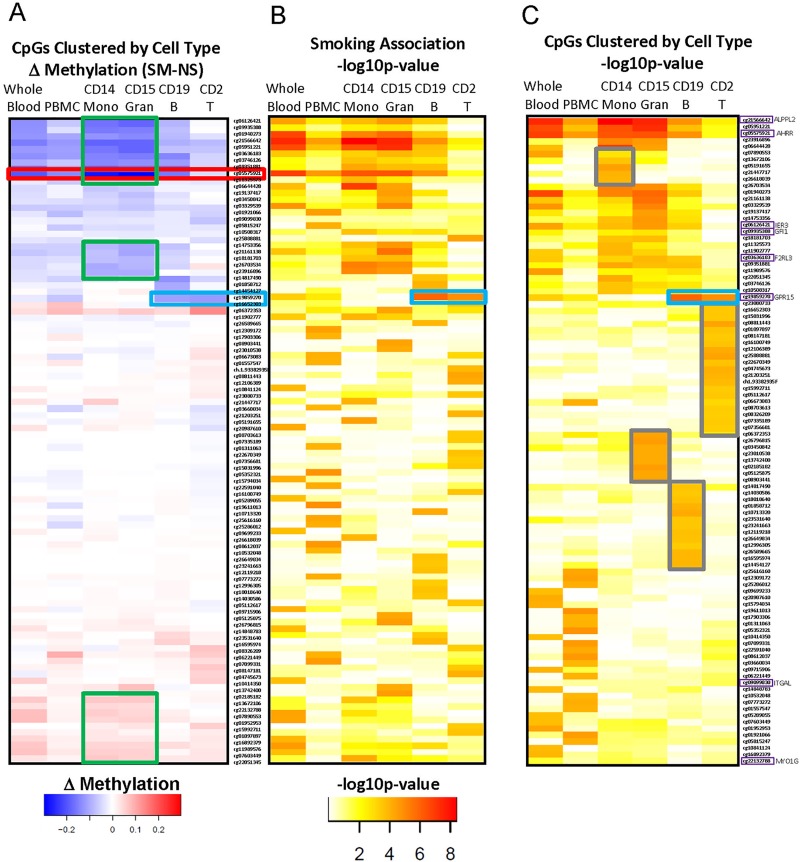
Clustered significant CpGs based on methylation effect size and significance in individual cell types. (A) Larger smoking effect sizes in myeloid cells (monocytes and granulocytes, green boxes) drive methylation effect size in whole blood (column one). (B) p-values for (A) show that significant CpG changes in whole blood are not always observed in PBMC (column two), although GPR15 is an exception to this (blue box). (C) Clustering CpGs by similarity of p-value patterns among cell types reveals cell-type specific smoking-associated CpGs (grey boxes). Most of these are not significant in PBMC or whole blood.

**Fig 3:**
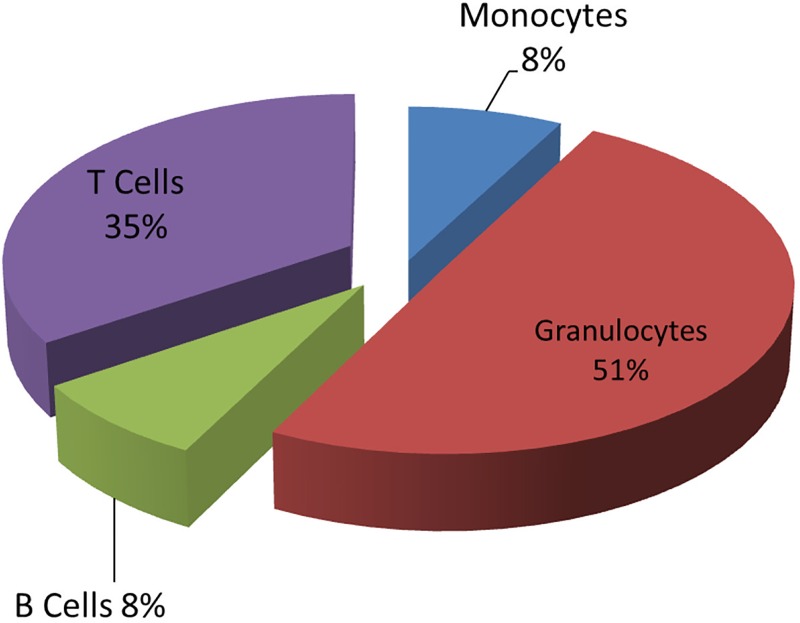
Distribution of major leukocyte cell types in whole blood.

To understand cell type-specific contributions to smoking-associated methylation changes measured in whole blood DNA, we used a second study population to examine methylation in purified CD14+ monocytes, CD15+ granulocytes, CD19+ B cells, CD2+ pan T cells, PBMCs and whole blood isolated from the same subject (see Table 2 for complete blood counts). Because this sample of 34 individuals was underpowered to assess genome-wide smoking associations, we focused on patterns of response to smoking. We profiled the 20 most significant CpG sites in each cell type and clustered the results by similarity of methylation changes among the 4 cell types (delta methylation, Fig 2A and 2B). The CpGs that showed the greatest smoking-associated methylation change in whole blood generally showed significant change in monocytes and granulocytes (Fig 2A, green boxes), and often also in B cells. The myeloid cell types represent a majority of leukocytes (Fig 3). The best example of this is *AHRR* cg05575921 (Fig 2A, red box). The strongest lymphoid-specific effect was observed for *GPR15* cg19859270 (Fig 2A, blue box). After clustering the same CpGs by similarity in p-value pattern (Fig 2C), distinctive groups of cell type-specific responses become apparent (Fig 2C, grey boxes). Importantly, the smoking-associated CpGs that were significantly detected in only one cell type, for example B and T cells (far right columns, Fig 2C), typically did not reach significance in whole blood or PBMCs.

**Fig 4:**
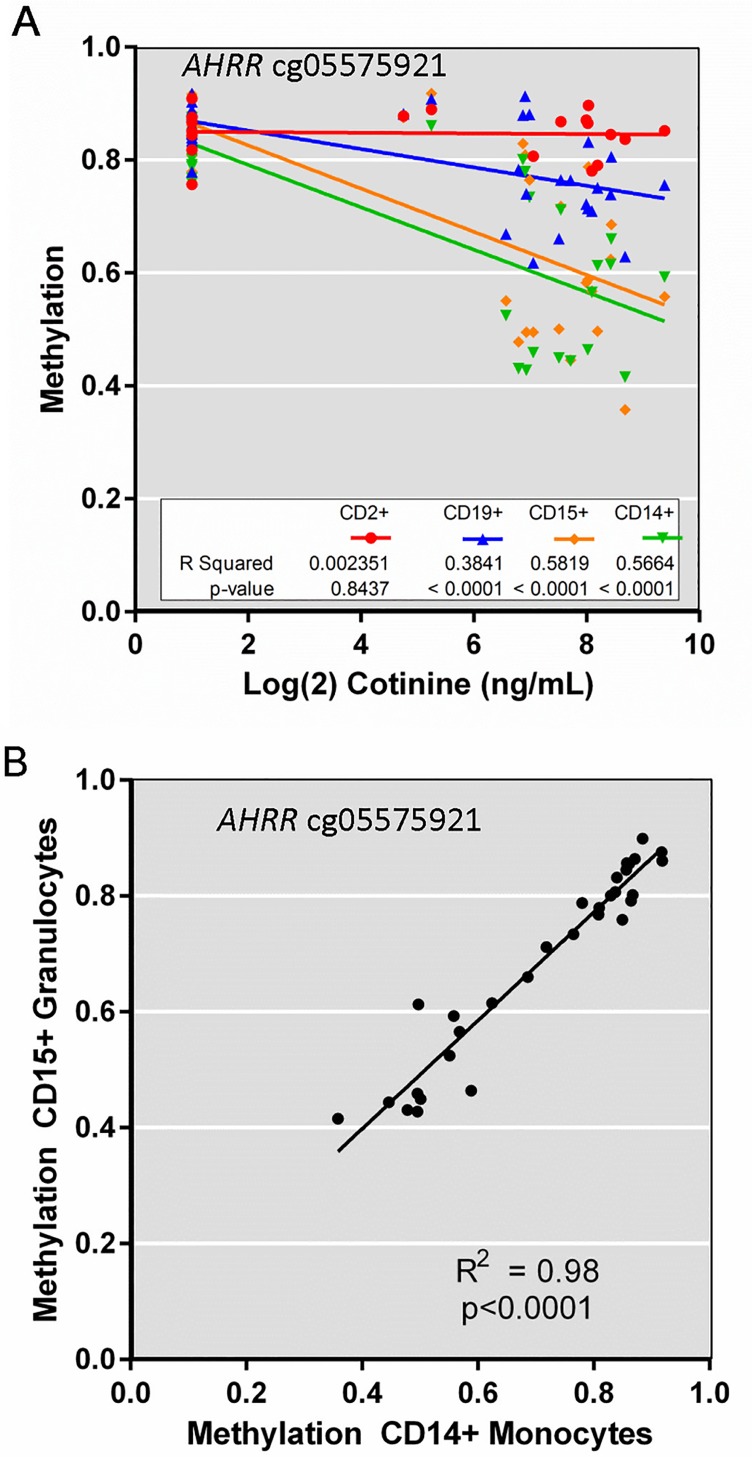
Effects of current smoking on *AHRR* cg05575921 methylation among monocytes, granulocytes, B cells and T cells. (A) Log2-transformed cotinine levels (ng/ml) were regressed with methylation beta-value measured for cg05575921 in each blood cell type. (B) Strong concordance of DNA methylation at cg05575921 (*AHRR*) between CD14+ monocytes and CD15+ granulocytes within individual subjects.

**Fig 5:**
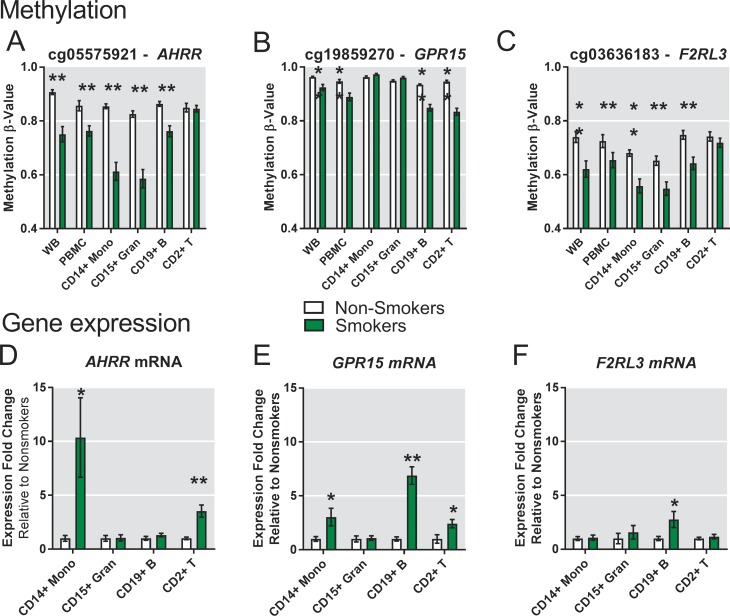
Comparison of smoking-associated CpG methylation (A-C) and gene expression (D-F) differences between cell lineages. Methylation levels were measured on 450K arrays and averages for nonsmokers and smokers are displayed (A) cg05575921 (*AHRR*), (B) cg19859270 (*GPR15*) and (C) cg03636183 (*F2RL3*). Gene expression level (D) *AHRR*, (E) *GPR15* and (F) *F2RL3* for each cell type was measured by RT-PCR and averages are shown based on smoking status. Expression is represented by fold change difference between smokers relative to the average of nonsmokers after normalization to β-actin. Bar = Mean ± Standard Error; *p≤0.05, **p≤0.001, Student’s t-test.

To examine differences among cell types in a more quantitative way we focused on a set of 8 CpGs, in or near 8 genes, that were highly significant in the EWAS analysis (circled on Fig 1A, boxed purple on Fig 2C, far right). Fig 4A compares serum cotinine levels with DNA methylation for *AHRR* cg05575921 for each cell type. We observed significant linear correlations in CD14+ monocytes (p = 7.61x10^-6^), CD15+ granulocytes (p = 1.13x10^-5^), and CD19+ B cells (p = 2.47x10^-5^), but not CD2+ T cells. DNA methylation levels for cg05575921 in monocytes vs granulocytes were strongly correlated within individual subjects (r^2^ = 0.9657, p<0.0001) (Fig 4B). Fig 5A shows that *AHRR* cg05575921 methylation levels were significantly reduced in whole blood, PBMCs, and hypomethylation was most pronounced in CD15+ granulocytes (-24%, p = 1.4x10^-6^) and CD14+ monocytes (-24.2%, p = 5.5x10^-7^). CpGs near *F2RL3*, *IER3*, *GFI1* and *ALPPL2* showed significantly reduced methylation in whole blood (Fig 5C, S3A and S3B Fig, and S3I Fig) but the difference was less pronounced in PBMCs. Similar to *AHRR* cg05575921, the profiles of change for each of these CpGs showed reduced methylation in CD14+ monocytes, CD15+ granulocytes and B cells, but showed no significant effect in T cells. Smoking-associated DNA methylation profiles for *GPR15*, *MYO1G*, and *ITGAL* (also called CD11A) (Fig 5B, S3C and S3D Fig) were unique. *GPR15* methylation level in smokers was significantly lower in PBMC (p = 0.0021) and whole blood (p = 0.0091), did not differ in myeloid lineage (CD14+ and CD15+, Fig 5B) but was strongly affected in B cells (-8.4%, p = 9.9x10^-7^) and T cells (-11.3%, p = 1.4x10^-6^). *MYO1G* displayed significantly increased methylation only in CD14+ monocytes (S3C Fig), while *ITGAL* cg09099830 showed significant differential methylation only in CD2+ T cells (S3D Fig).

### Reduced Representation Bisulfite Sequencing

**Fig 6:**
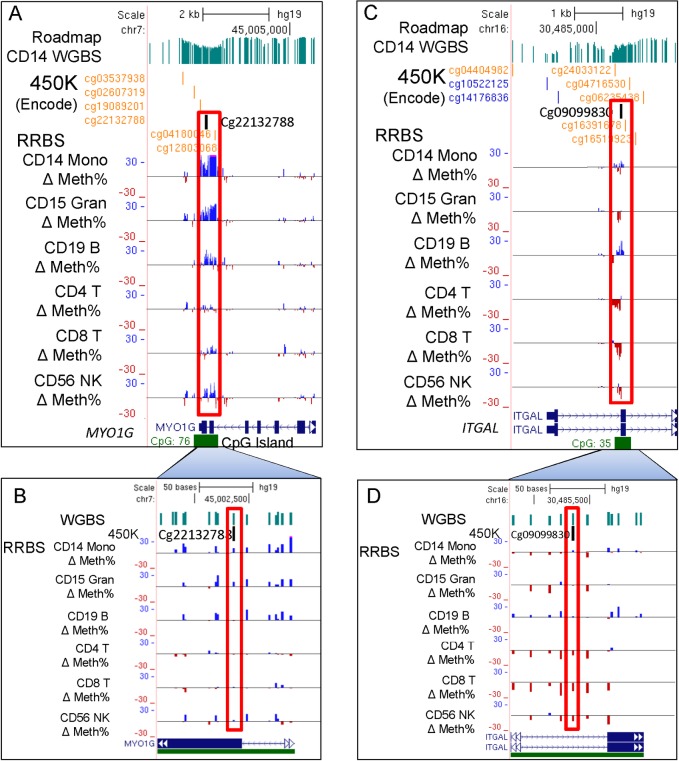
RRBS demonstrates regional impact and lineage-specific smoking DMRs within *MYO1G* and *ITGAL*. (A) Increased levels of CpG methylation were observed in *MYO1G* across a ~500-bp region with greater effects in myeloid cell types. (B) At cg22132788 the pattern of change across cell types closely matches 450K array results (S3C Fig). (C) RRBS reveal hypomethylation in B cells that was not detected in 450K results. (D) T cell subsets show loss of methylation consistent with 450K array results.

To fine map the regions near smoking-associated CpGs and identify DMRs we carried out RRBS analysis of DNA from monocytes, granulocytes, B cells and T cell subsets (CD4, CD8, CD56 natural killer cells) as described previously for *AHRR* [31]. Limitations of the RRBS library technique (i.e. the necessity of nearby MspI sites) permitted examination of only *MYO1G*, *ITGAL*, *F2RL3*, and *ALPPL2*. Comparing differentially methylated CpGs in the 3’ end of *MYO1G* across cell types on the genome browser (Fig 6A, red box), we observed a ~500-bp RRBS DMR displayed increased methylation (blue bars above line represents increased methylation) in monocytes, granulocytes and B cells. At higher magnification (Fig 6B, red box) methylation profiles at cg22132788 determined by RRBS closely match the 450K profiles. The *ITGAL* DMR shows up strongly in CD4 and CD8 T cell subsets (Fig 6C, red box), which is consistent with the 450K CD2 pan T cell result, while RRBS reveals that CD19 B cells display smoking-associated hypermethylation at this locus, which was not detected by 450K array. Supporting S4 Fig shows that DMRs in *F2RL3* and near *ALPPL2* displayed groups of CpGs that were consistently hypomethylated across all cell types in smokers. Interestingly, the DMR at cg21566642 near *ALPPL2* maps to a long noncoding RNA (lncRNA), AC068134.

### Heterogeneity of smoking-associated gene expression across blood cell types

To examine the relationship between DNA methylation and gene expression among cell types, we measured mRNA levels using RT-PCR for the 8 coding genes nearest these CpGs in 4 cell types (Fig 5D–5F, S3E–S3H Fig). *ALPPL2* gene expression was undetectable. Although multiple cell types from smokers showed reduced methylation at cg05575921, *AHRR* gene expression was most strongly upregulated in CD14+ monocytes (FC = 10.3, p = 0.021) and this was correlated with cotinine levels (S6A Fig). *AHRR* was also upregulated in CD2+ T cells (FC = 3.5, p = 0.00027) (Fig 5D). *AHRR* gene expression and cg05575921 methylation displayed a strong inversely correlation in monocytes (r^2^ = 0.59, p<0.0001, S6B Fig), but were uncorrelated in CD2+T cells (p = 0.741), CD19+ B (p = 0.330) or granulocytes (p = 0.578) among all subjects. *GPR15* expression was significantly up-regulated in CD14+ monocytes (FC = 3.0; p = 0.02), CD19+ B (FC = 6.8; p = 1.17x10^-6^), and CD2+ T cells (FC = 2.4; p = 0.016) (Fig 5E). *F2RL3* was significantly up-regulated only in CD19+ B cells (FC = 2.7; p = 0.03) (Fig 5F). *IER3*, *GFI1*, *MYO1G* and *ITGAL* mRNA were all easily detected but did not display significant differences (S3H Fig). Of note, IER3 expression was dramatically increased in 3 smokers (>20-fold) but not at all in most smokers (S6C Fig).

### Chromatin state, DMRs and gene expression

Histone modifications (Roadmap Project[33]) and transcription factor (TF) occupancy (ENCODE ChIP-seq,[34]) were examined for selected RRBS analyzed genes and cell types (S4 Fig, S5 Fig). For *F2RL3*, the DMR (S4A Fig, red box) was adjacent to strong H3K27ac/H3K4me1 peaks in B cells. This is suggestive of an active enhancer in the promoter, however, the same region also displays a strong repressive H3K27me3 mark (S4A Fig, blue box) and gene expression was very low in unexposed B cells. Other cell types show similar chromatin status in this region. Thus *F2RL3*, despite the presence of impressive DMRs in all cell types and abundant possible TF binding, remains repressed at the gene expression level. Similarly, the DMR in the potential enhancer near *ALPPL2* (and lncRNA AC068134) is also strongly marked by H3k27me3 (S4B and S4C Fig, blue box). In contrast, *MYO1G* and *ITGAL* (CD11A), show strong activating histone modification (H3K4me3, H3K4me1, H3K27ac) with no H3K27me3 marks at their promoters and near the DMRs (S5 Fig). These genes show abundant TF binding (dark boxes) near their promoters in lymphoid cells (indicated by “G”), particularly ELF1, EGR1, NFKB, PU.1, PAX5 and MEF2A in *MYO1G* and EBF, PU.1 and EGR1 in *ITGAL*.

### Smoking-associated changes in cell-type composition and cell-type specific effects

Smoking can strongly affect immune cell composition [21,35] although in our small study we observed only modest, nonsignificant differences between nonsmokers and smokers (based on either computation or complete blood counts, Tables 1 and 2, S2A–S2F Fig). Relative leukocyte composition (Fig 3) in relationship to relative change in cell-type composition and cell-type specific methylation effects may combine to produce different measured outcomes in whole blood. We observed that *AHRR* had greatly reduced methylation in granulocytes and monocytes which compose ~60% of leukocytes, and granulocytes are frequently observed to increase in smokers [21], making the smoking-associated methylation effect easily detected in whole blood (Fig 5A). In contrast *ITGAL* methylation difference was most pronounced in T cells (Fig 6B, S3D Fig), which often display reduced proportions in the blood of smokers [21], and significant methylation change was not detected in whole blood.

## Discussion

We examined methylation levels in a relatively heavy smoking population and followed up with leukocyte cell-type analysis to assess methylation change patterns relative to whole blood. It is remarkable that 28 of the top 30 genome-wide significant CpGs have previously been observed in one or more studies (S1 Table), indicating an extraordinary level of reproducibility of the smoking effect on the immune system, irrespective of population sampled. In the present work, the distribution of males and females is skewed, however, despite this limitation and the small sample size in the cell type study, the results align well with other studies. That we observe highly significant differences for selected CpGs in both whole blood and in isolated cell types from a small population sample further indicates the robust nature of the smoking-DNA methylation biomarker. Interestingly, despite relatively heavy smoking levels observed, pack-year analysis identifies many fewer (38 CpGs) genome-wide significant CpGs than “any smoking” (738 CpGs). Although *AHRR* cg05575921 remains the most significant smoking-associated CpG in pack-year analysis, higher levels of smoking showed only a modest increase in effect size (see Fig 1B and 1C, S1A and S1B Fig). This lack of dose-response at higher exposures suggests the smoking-methylation effect is saturated due to a relatively large reservoir of blood cells, such as T cells, that remain largely unaffected. In contrast, *AHRR* cg05575921 and many other CpGs appear very sensitive to relatively light smoking levels over a short period of time (S1B Fig), which is consistent with other studies [18] [19]. *AHRR* methylation and gene expression in monocytes were strongly correlated with serum cotinine, and also with each other (S6A and S6B Fig). We observed that some individuals were outliers when comparing cotinine levels with either methylation, or gene expression (S6A Fig), and this is likely due to new sources of nicotine exposure, such as electronic cigarettes. Although no subjects reported electronic cigarette usage, the use of electronic cigarettes which deliver pure nicotine without combustion smoke is very common now. It clearly has potential to confound the smoking-nicotine-cotinine relationship and this should be considered in future studies of smoking.

Among smoking-associated CpGs, we observed strong enrichment for ontological pathways related to hematological cancers, cardiovascular system disease, hematopoietic system disease, and nervous system cancer (Table 1, S3 and S4 Tables). Of interest, most of these disease-associated genes displayed DMRs, with multiple, grouped CpGs that differ by smoking (e.g. *AHRR*, *ANPEP*, *GFI1*, *HOXA9*, *HOXA13*, *HOXB*, *HOXD11*, *IER3*, *ITGAL*, *RARA*, *RARG*, *RUNX3*, and *SUMO1*). *GFI1*, *HOXA*, *HOXB*, *RARA* and *RUNX3* are central to transcriptional circuits important in malignant hematopoiesis [36–38]. We observe hypomethylation of six CpG sites in *ITGAL* (Integrin alpha L chain, CD11a) in whole blood, and we can attribute this primarily to a T cell-specific response (e.g. cg09099830, S3D Fig; see RRBS DMRs shown in S5D Fig).

Chronic long-duration, high-intensity smoking could affect many aspects of the immune system and smokers with many years of heavy smoking might display altered methylation suggestive of chronic disease pathology. We used a novel stratified analysis to identify CpGs associated with years of smoking among heavier smokers (≥22 cigarettes/day) relative to lighter smokers (<22 cigarette/day). Tumor-associated genes, *MTSS1*, *NKX6-2*, and *BTG2* had CpGs that were highly significant in heavy smokers but showed little or no significance in light smokers (S4 Table). Metastasis Suppressor 1 (*MTSS1*), is inactivated in metastatic cancer [39] and has prognostic value for breast cancer [40] and lung cancer [41]. *NKX6-2* and *BTG2* (B-Cell Translocation Gene 2) are candidate tumor suppressor genes [42,43]. Further analysis of *MTSS1*, *NKX6-2*, and *BTG2* in the blood of heavy, long-time smokers might reveal a relationship between methylation and early signs of pathology or disease.

Characterizing cell-type differences in the epigenetic response to tobacco smoke is important for understanding the variable detection of DMRs in whole blood DNA and for interpretation of the biological significance of methylation differences. Comparing methylation in whole blood to PBMC, and to individual cell types (e.g. Figs 2A–2C, 5A, 5C, S3A–S3C Fig), we can conclude that effects that are unique to granulocytes or myeloid lineages will be more easily detected in whole blood rather than PBMCs. This is because granulocytes are greatly depleted during PBMC preparation (S2B Fig). Several of the CpGs we examined (e.g. *AHRR* cg05575921, *ALPPL2*, *IER3*, *F2RL3*, *GFI1* and *MYO1G*) showed greater methylation differences in myeloid lineages (CD15+ granulocytes, CD14+ monocytes) than in lymphoid lineages. In addition, most other strong differentially methylation CpGs in whole blood (Fig 2A green box) had a similar pattern. This methylation pattern across cell types suggests a common underlying biological mechanism for altering these CpGs, possibly related to innate immune response and cell-type proliferation rate. Methylation levels were strongly correlated between myeloid lineages, suggesting that smoking may have effects at the level of myeloid progenitor cell differentiation or alter regulation of common lineage-specific transcription factors.

The mechanism for tobacco smoke-induced effects in lymphoid lineages is likely to be very different than myeloid effects. *GPR15*, previously implicated in the T cell inflammatory response [44], has a unique lymphoid lineage-specific pattern for both methylation (cg19859270) and expression (Figs 2, 5B and 5E). It was recently suggested that the smoking-associated *GPR15* methylation signal in whole blood [45] was due to an increased number of a specific T-cell subtype that expresses GPR15 protein on its cell surface. The present data are consistent with this observation for T cells although we also detect significant changes for both *GPR15* methylation and expression in CD19+ B cells, which were not measured by Bauer et al. An increased number of *GPR15* expressing T and B cells may represent a lymphoid-specific inflammatory response to tobacco smoke that originates at the lymphoid progenitor cell level or in response to inflammatory signals generated from injured tissues. Similar to *GPR15*, *ITGAL* methylation differences were strongest in T cells (Fig 6C, S3D Fig), which were somewhat lower in smokers, reducing the effect size in whole blood (S3D Fig). In contrast, the effect size of the *AHRR* methylation difference could be amplified by the increased proportion of granulocytes observed in smokers. Exposure-related shifts in cell-type percentage in whole blood might obscure or positively influence detection of exposure- or disease-associated methylation effects in whole blood. Smoking may induce activation of many different cell types in whole blood and this activation is not captured in the current separation scheme and approach to cell type composition deconvolution. We are currently measuring smoking-altered methylation in an expanded set of separated leukocyte cell types and extending this concept further using mass cytometry-based immuno-phenotyping to identify uniquely affected cells among >20 leukocyte subtypes [46].

Lineage-specific methylation changes may result from many possible biological mechanisms. Wiencke et al [23] have suggested that differences might be driven by immune cell activation (e.g. activated natural killer cells) and clonal selection in smokers. A more direct type of selection may involve selection for progenitor cells in the bone marrow that express genes that permit hematopoietic differentiation under conditions of toxic exposure to carbon monoxide, DNA damaging polycyclic aromatic hydrocarbons, nicotine and many thousands of tobacco smoke components. Chromatin state, transcription factor binding and methylation level vary dramatically across hematopoietic lineages at thousands of enhancers and methylation level at these locations is negatively correlated with enhancer activity [27,47]. It is noteworthy that the longest lived leukocyte cells in the circulation (B and T cells), which receive the greatest cumulative dose of tobacco smoke, appear to be less affected by smoke exposure relative to the short-lived myeloid lineages.

RRBS analysis of several of the smoke-altered CpGs across cell types validated the 450K results (Fig 6) and permitted striking visualization of DMRs (100-500nt) that co-locate with individual affected CpGs. Fine mapping by RRBS revealed co-location of DMRs with actively repressed enhancers in *F2RL3* and near *ALPPL2* (S4 Fig) and active enhancers in *MYO1G* and *ITGAL* (S5 Fig). These enhancers co-locate with numerous occupied transcription factor binding sites in hematopoietic cells as determine by ENCODE ChIP-seq (S4 and S5B and S5C Figs, cell line code G Fig). The observed relationship between altered methylation and gene expression among leukocyte subtypes suggests that cell-type context is important for interpreting biological implications. Each of the smoking-associated CpGs are located near or in ENCODE/Roadmap identified enhancer regions displaying H3K4me2/H3K27Ac marks, DNaseI hypersensitivity, and/or clusters of transcription factor binding sites in hematopoietic cells (e.g., S4A–S4C and S5A–S5E Figs). For example, ENCODE data shows that the *ITGAL* DMR displayed strong binding of the B cell lineage-commitment factor EBF1 [48] in B lymphoid cells (S5E Fig red box). In S5D Fig the DMR in CD19+ B cells showed increased methylation with smoking while each of the other cell types showed strong demethylation.

Changes in DNA methylation across a DMR region could reflect changes in transcription factor binding and chromatin state, and one might hypothesize that such DNA methylation changes would be accompanied by gene expression changes. However, it is notable that there are distinctive lineage-specific smoking-response profiles for gene expression that do not correspond to distinct DNA methylation profiles (Fig 5, S3 Fig). For example, *AHRR* cg05575921 methylation was reduced in both myeloid lineages and B cells—but not T cells (Fig 5A). However, differential mRNA expression was strong in monocytes, no change in granulocytes, and surprisingly, also showed increased expression in both B and T cells (Fig 5D). Thus while the usual assumption about the inverse relationship between enhancer methylation and mRNA expression appears to hold for monocytes, the situation is more complex in different cell-type contexts. It is possible that in response to exposure, a subset of CD14+ monocytes such as CD16+ monocytes, and also a subset of CD2+ T cells such as activated natural killer (NK) cells, have de-methylated *AHRR*, as suggested by Wiencke et al [23], leading to up-regulated *AHRR* mRNA. The dynamic range of real-time PCR detection of *AHRR* mRNA is such that upregulation in even a small number of activated cells would be detected much more easily than differences in methylation level. *F2RL3* also displayed highly significant decreases in methylation level across monocytes, granulocytes, B cells and T cell subsets (Fig 5C, S4A Fig), but expression changes were only evident in CD19+ B cells of smokers (Fig 5F). Roadmap Project [33] histone modifications and RNA-seq data for *F2RL3* gene were available for nonsmoking individuals. The presence of H3K27me3 marks and undetectable RNA-seq (S4A Fig, blue box) in B cells strongly suggests active repression of the *F2RL3* gene. Similarly, the strong DMR in all cell types located near *ALPPL2* also displayed strong repressive histone marks in all cell types (S4C Fig, CD19 shown, blue box). It is interesting that *IER3* (Immediate early response 3), which is known to be induced by growth factors, cytokines, ionizing radiation, or viral infection [49] was observed to have extremely high induction of gene expression in B cells of 3 individual smokers (S3E and S6C Figs) but this did not reflect methylation levels in these individuals or smoking dose, and was not associated with any variable identified on our medical history for these participants. Lineage-specific transcription factors/co-activators and chromatin conditions, as well as genetic variability may mediate many of these differences in the methylation/gene expression responses across cell types and individuals.

Characterizing the distinct exposure-induced DNA methylation patterns in immune cells may provide an early view of immune system dysfunction that might predispose to disease. In addition, developing more detailed models to assess the specific leukocyte subtypes affected by exposure could be important for understanding the mechanism driving methylation change in hematopoietic cells and the subsequent functional consequences. While functional links between methylation and gene expression may not be apparent in a cross-sectional study, exposure-induced DMRs in immune cells might alter enhancers in a way that affects subsequent responses and leads to pathology. For example, we hypothesize that the coordinated change in *AHRR* methylation and gene expression in CD14+ monocytes obtained from smokers may point to a subset of monocytes primed to overcome a smoking-induced block to differentiation, either caused by DNA damage or by Ah Receptor activation-mediated suppression of monocyte-to-macrophage differentiation [50]. As recently reported, *AHRR* mRNA was strongly up-regulated when normal monocytes were induced to differentiate into macrophages [51] and the *in vivo* smoking-associated changes in *AHRR* that we observe may represent the beginning of this transition. It is unknown if these smoking affected monocytes/macrophages would display an altered cellular phenotype, such as a proinflammatory response, but we have hypothesized that these cells could be implicated in atherosclerotic plaque formation related to monocyte-derived endothelial macrophages [31].

Understanding the meaning of smoking-associated epigenetic changes remains a challenge. Although many studies have explored smoking and DNA methylation using whole blood or mononuclear cells, the present study provides a detailed look at selected CpGs in granulocytes, monocytes, B and T lymphocytes, and reveals their genomic context within fine-mapped DMRs. Importantly, we demonstrated cell-type specificity of smoking-induced methylation changes and reveal discordance of gene expression changes complicating biological interpretation. More detailed studies are needed to fully characterize smoking effects on the whole epigenome, to evaluate how genotype may influence epigenetic alterations and to reveal mechanisms that link these factors with cellular phenotypes and biological outcomes.

## Methods

### Study populations

253 individual study participants consisting of 172 smokers and 81 nonsmokers enrolled between 1993 and 1995 as healthy volunteers from the general public in Durham and Chapel Hill, North Carolina. These subjects were part of a community-based sample comprised of 294 healthy unrelated blacks and whites; collection and processing have been described in several studies [3,4,52]. An independent group of black and white females (20 smokers and 14 nonsmokers) was recruited at the NIEHS Clinical Research Unit (protocol 10-E-0063) between March 2013 and January 2015 from the Raleigh, Durham and Chapel Hill, NC area. All nonsmokers were self-reported as not having smoked >100 cigarettes in their lifetime. Smokers reported their average daily cigarette consumption for the past 3 months. Age and smoking history of all subjects are given in Tables 1 and 2 as average and range. Peripheral blood monocytes, lymphocytes, eosinophils, basophils and neutrophils were counted by an automated cell counter, Coulter HmX AL Hematology Analyzer (Beckman Coulter, UK) by the Hematological Laboratory at NIEHS. Serum nicotine/cotinine levels were measured by HPLC-MS (Quest, Inc). More details for all methods are provided in the Supporting files.

### Ethics statement

The Institutional Research Board of the National Institute of Environmental Health Science-NIH approved this research. Written informed consent was obtained from all subjects and the analysis of samples was carried out under approved human subject protocols (NIEHS 86-E-0037 and 10-E-0063).

### Peripheral blood leukocyte subtype isolation

Granulocytes were isolated directly from whole blood using anti-CD15+ antibody–coated magnetic beads following the protocol (Invitrogen). Density gradient centrifugation using Histopaque-1077 Ficoll medium and Accuspin™ Tubes (Sigma-Aldrich) was used to isolate the mononuclear layer, which was used for isolation of CD14+ monocytes, CD2+ pan T lymphocytes, CD19+ B lymphocytes, CD4 T cells, CD8 T cells and CD56 natural killer cells using antibody-coated magnetic beads (Invitrogen).

### Methylation analyses

Extracted DNA was bisulfite converted and applied to the Human Methylation 450 BeadChip (Illumina) to measure methylation at 485,577 CpG sites. The ChAMP pipeline was used to normalize and batch correct methylation array data [53–55]. Probes with SNPs (MAF > = 0.01 in 1000 Genomes Project) at CpG sites were excluded to avoid SNP (single-nucleotide polymorphism) effects on methylation measurement. To investigate associations between smoking and DNA methylation, normalized and batch-corrected beta-values were transformed to log ratio, defined as log_2_[β/(1 –β)], and then fitted using robust linear regression [32] adjusted for age, sex, race and cell type counts ("CD4T", "CD8T", "Bcell", "Mono", "NK", "Neu", "Eos"), estimated using the method of Houseman et al. [22]. The difference in methylation level between groups was calculated by t-test. To explore the associated between years of smoking in heavy smokers, we considered the top 1000 CpGs associated with any level of smoking, then we carried out a stratified analysis based on the median level of smoking, <22 cigarettes/day versus ≥22 cigarettes/day, and then ranked CpGs by p-value for association with years of smoking in each stratum. We then calculated the difference in rank across the strata for each CpG.

### Reduced Representation Bisulfite Sequencing

To investigate the relationship between smoking and CpG methylation not captured by microarray, and to qualitatively visualize DMRs, RRBS libraries were constructed as previously described [20,31] (see supporting Methods). Briefly, libraries were constructed from DNA extracted from CD14+ monocytes, CD15+ granulocytes, CD19+ B cells, CD4+ T cells, CD8+, and CD56+ NK cells from 5 smokers and 5 nonsmokers and sequenced on Illumina HiSeq 2500 at the NIH Intramural Sequencing Center.

### Enrichment analysis of methylation regions associated with smoking

We used the GREAT (Genomic Regions Enrichment of Annotations Tool, http://bejerano.stanford.edu/great/public/html/) [47] to find enriched functional terms of genes near our top 738 CpGs as these terms indicate the potential regulatory functions of these CpGs. Each CpG probe was first assigned a gene regulatory domain that extends in both directions to the midpoint between the nearest gene's TSS and the nearest adjacent gene's TSS, but no more than 100kb in one direction; then GREAT was run with default parameters. We focused on the enrichments having the following properties: (1) regions hits > 100; (2) hypergeometric test enrichment fold > 2; and (3) FDR q < 0.05. If a term was a parent of another term based on the ontology tree, then the parent term was removed.

### Reverse transcription quantitative polymerase chain reaction (RT-qPCR)

RNA and DNA were isolated using the ALLPrep DNA/RNA/miRNA Universal Kit (Qiagen). cDNA was generated using the SuperScript® III First-Strand Synthesis (Life Technologies). For each individual RNA sample, target and reference genes (*AHRR*, *GPR15*, *F2RL3*, *IER3*, *GFI1*, *ITGAL* and ß-actin) were amplified in triplicate using TaqMan assays designed to span exon junctions (Life Technologies AHRR: Hs01005075_m1; GPR15: Hs00922903_s1; β-actin: Hs01060665_g1; F2RL3: Hs01006385_g1; ALPPL2: Hs00741068_g1; IER3: Hs04187506_g1; GFI1: Hs00382207_m1), using the Universal PCR Master Mix (Life Technologies) and the ABI 7900HT Real-time PCR machine. Gene level data was normalized to β-actin and fold change (FC) was assessed relative to nonsmokers using delta-delta Ct method. AHRR expression levels in CD14+ monocytes for 10 individual subjects were previously reported [31].

## Supporting Information

S1 Fig Modest impact of higher smoking dose or cumulative smoking dose on methylation of *AHRR* cg05575921. (A) Scatterplot of cg05575921 methylation versus cigarettes per day. (B) Scatterplot of cg05575921 methylation versus pack-years (packs smoked per day x years of smoking).
(TIF)

S2 Fig The cell composition (%) of each collected cell fraction from nonsmokers and smokers was determined computationally by cell-type specific methylation signature using Housman et. al [22] and Reinius et al [25]. The estimated composition of CD14+, CD15+, CD4+, CD8+, CD19+, CD56+ in (A) whole blood, (B) Peripheral blood mononuclear cells (PBMC), (C) CD14+, (D) CD15+, (E) CD19 and (F) CD2+T cells. PBMC fraction is nearly devoid of CD15+ granulocyte cells.
(TIF)

S3 Fig **Methylation profiles in whole blood, PBMC, and purified leukocytes**: (A) cg06126421 (*IER3*), (B) cg09935388 (*GFI1*), (C) cg22132788 (*MYO1G*), (D) cg09099830 (*ITGAL*) and (I) cg21566642 (*ALPPL2)*. Gene expression profiles in purified leukocytes: (E) *IER3*, (F) *GFI1*, (G) *MYO1G*, and H) *ITGAL*.
(TIF)

S4 Fig DMRs within *F2RL3* and near *ALPPL2* co-locate with gene regulatory regions but display repressive chromatin. (A) Decreased levels of CpG methylation were observed in *F2RL3* across a ~300-bp region in each cell type but the H3K27me3 mark and lack of expression suggests active repression. (B) At Cg21566642 near *ALPPL2* repressive H3K27me3 marks were observed.
(TIF)

S5 Fig Chromatin state, TF binding and transcription of *MYO1G* and *ITGAL*. (A) Increased levels of CpG methylation were observed in *MYO1G* across a ~500-bp gene regulatory region (B) with greatest increase in methylation in CD14+ monocytes. (C) The promoter of the highly expressed *MYO1G* gene displays activating histone marks and transcription factor binding. (D) *ITGAL*, sometimes called CD11A, shows a unique DMR in CD19+ B cells. It displays activating histone modifications and is highly expressed in CD19+ B cells. (E) EBF1, a B cell specific TF occupies its binding motif near the DMR in B lymphoblastoid cells.
(TIF)

S6 Fig Gene expression in *AHRR* and *IER3*. (A) *AHRR* mRNA expression (RT-PCR, log2 fold change relative to nonsmokers) in monocytes vs serum cotinine values. Cotinine outliers marked by red. (B) *AHRR* mRNA levels (log2 fold change) in monocytes vs methylation (log2) in monocytes. Cotinine outliers (red) were not outliers for methylation vs expression, suggestive of a secondary exposure to nicotine such as eCigarettes. (C) Individual *IER3* log2 fold change expression values in B cells for nonsmokers (n = 10) and smokers (n = 19) relative to nonsmoker mean.
(TIF)

S1 File Supporting Information Methods. (DOCX)

S1 Table Smoking associated CpG sites. (DOCX)

S2 Table GREAT Functional Analysis. (DOCX)

S3 Table GREAT Genes Enriched in Hematological Cancers. (DOCX)

S4 Table Top 100 CpGs associated with duration of smoking among heavy smokers. (DOCX)
