# Correction: Distinct Epigenetic Effects of Tobacco Smoking in Whole Blood and among Leukocyte Subtypes

**DOI:** 10.1371/journal.pone.0178308

**Published:** 2017-05-17

**Authors:** Dan Su, Xuting Wang, Michelle R. Campbell, Devin K. Porter, Gary S. Pittman, Brian D. Bennett, Ma Wan, Neal A. Englert, Christopher L. Crowl, Ryan C. Gimple, Kelly N. Adamski, Zhiqing Huang, Susan K. Murphy, Douglas A. Bell

The tenth author’s name is spelled incorrectly. The correct name is: Ryan C. Gimple.
