# Supplementary figures and images for: Distinct Epigenetic Effects of Tobacco Smoking in Whole Blood and among Leukocyte Subtypes
Source: PLoS One. 2016 Dec 9;11(12):e0166486. doi: 10.1371/journal.pone.0166486 (PMC5147832; doi:10.1371/journal.pone.0166486)

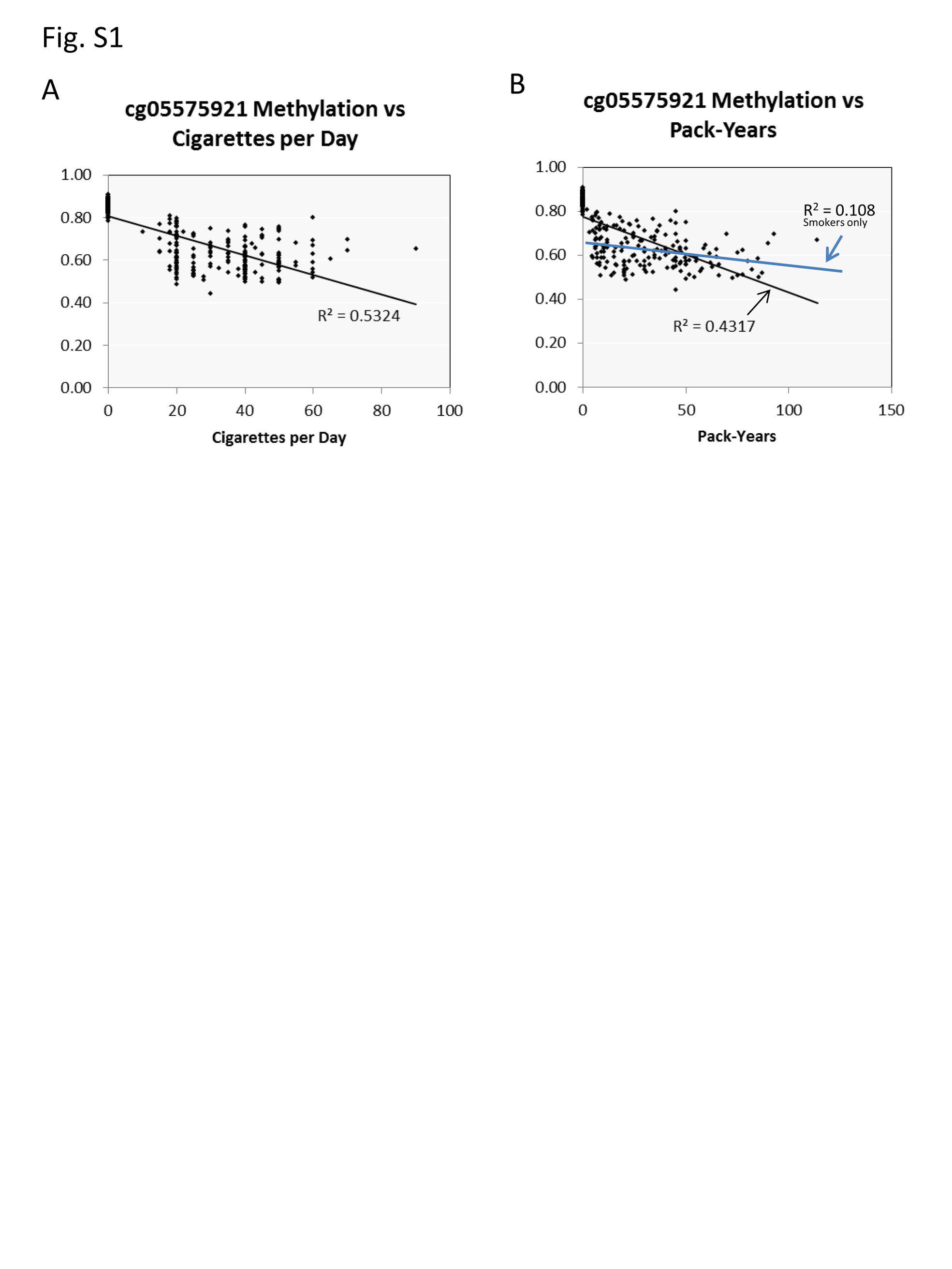

Supplement: S1 Fig — (A) Scatterplot of cg05575921 methylation versus cigarettes per day. (B) Scatterplot of cg05575921 methylation versus pack-years (packs smoked per day x years of smoking). (TIF) [file pone.0166486.s001.tif]

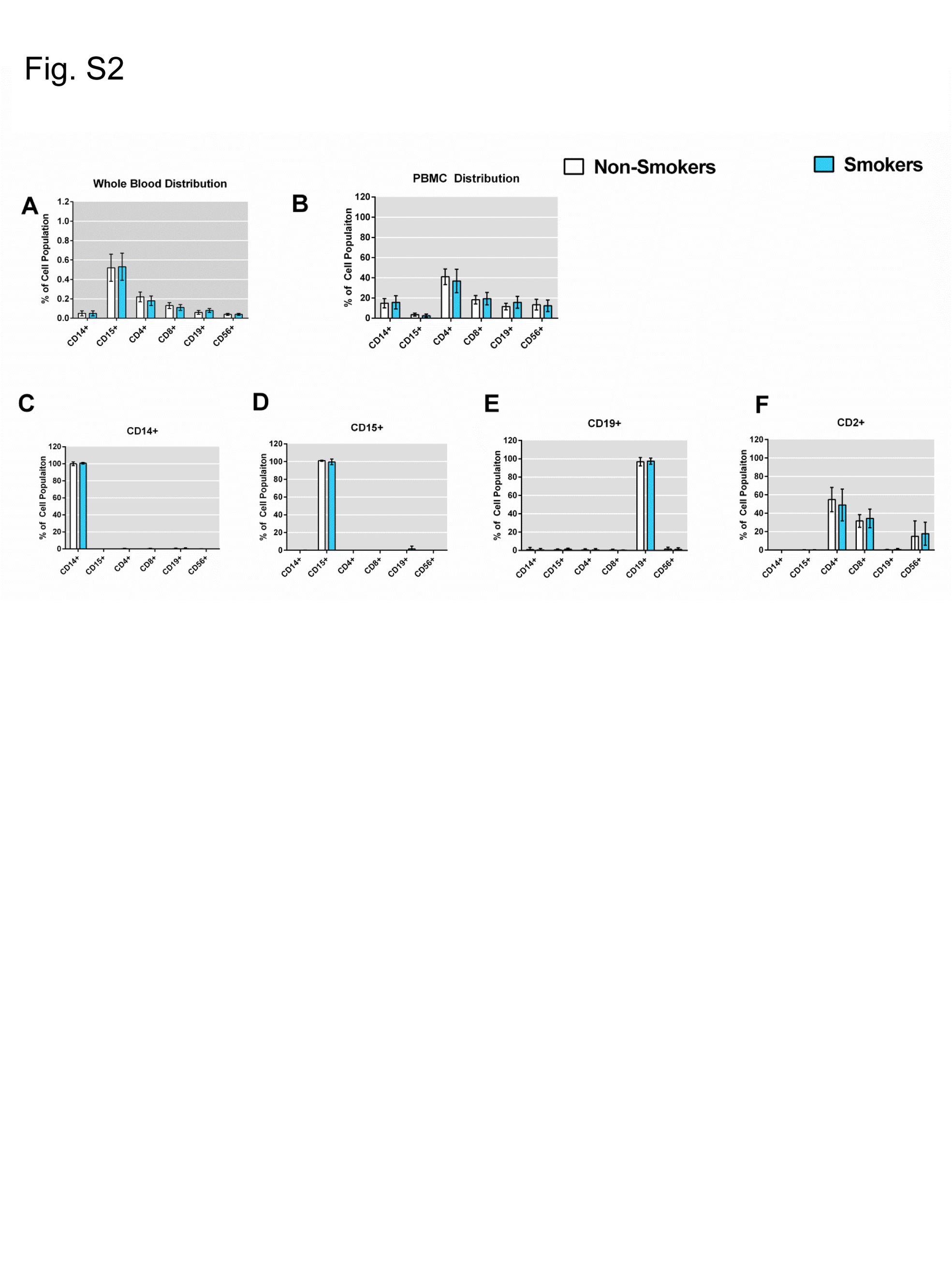

Supplement: S2 Fig — The estimated composition of CD14+, CD15+, CD4+, CD8+, CD19+, CD56+ in (A) whole blood, (B) Peripheral blood mononuclear cells (PBMC), (C) CD14+, (D) CD15+, (E) CD19 and (F) CD2+T cells. PBMC fraction is nearly devoid of CD15+ granulocyte cells. (TIF) [file pone.0166486.s002.tif]

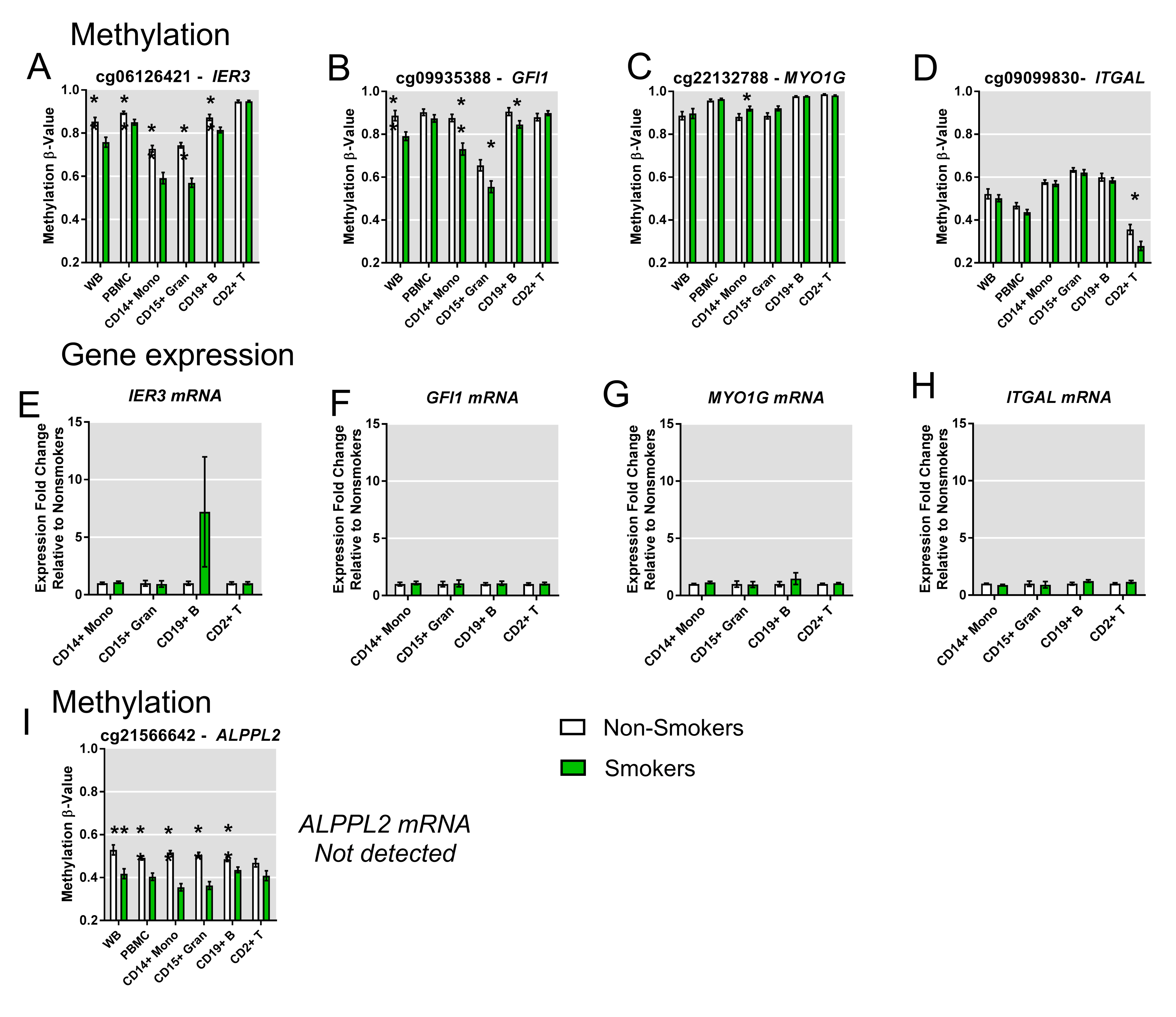

Supplement: S3 Fig — Methylation profiles in whole blood, PBMC, and purified leukocytes: (A) cg06126421 (IER3), (B) cg09935388 (GFI1), (C) cg22132788 (MYO1G), (D) cg09099830 (ITGAL) and (I) cg21566642 (ALPPL2). Gene expression profiles in purified leukocytes: (E) IER3, (F) GFI1, (G) MYO1G, and H) ITGAL. (TIF) [file pone.0166486.s003.tif]

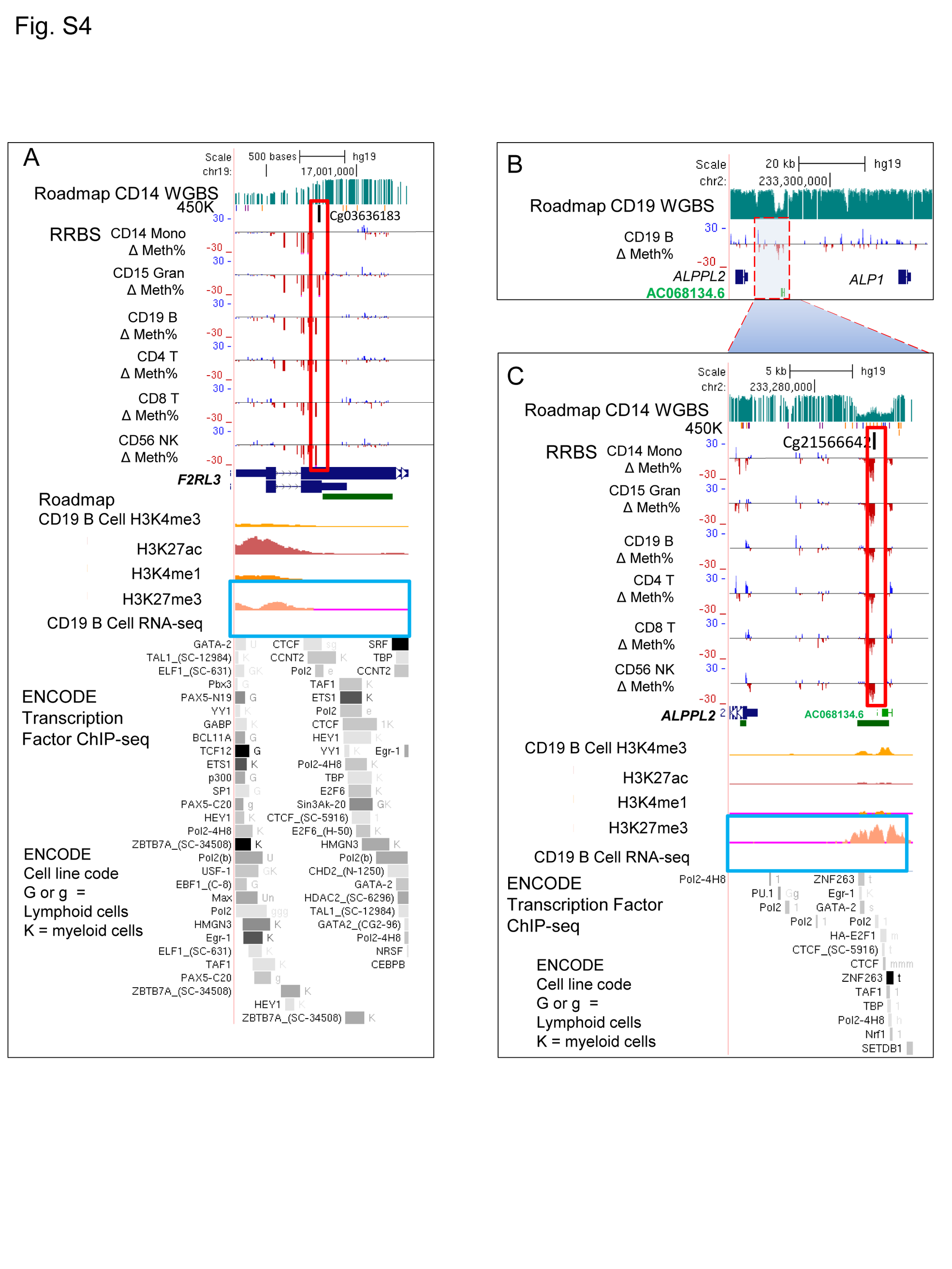

Supplement: S4 Fig — (A) Decreased levels of CpG methylation were observed in F2RL3 across a ~300-bp region in each cell type but the H3K27me3 mark and lack of expression suggests active repression. (B) At Cg21566642 near ALPPL2 repressive H3K27me3 marks were observed. (TIF) [file pone.0166486.s004.tif]

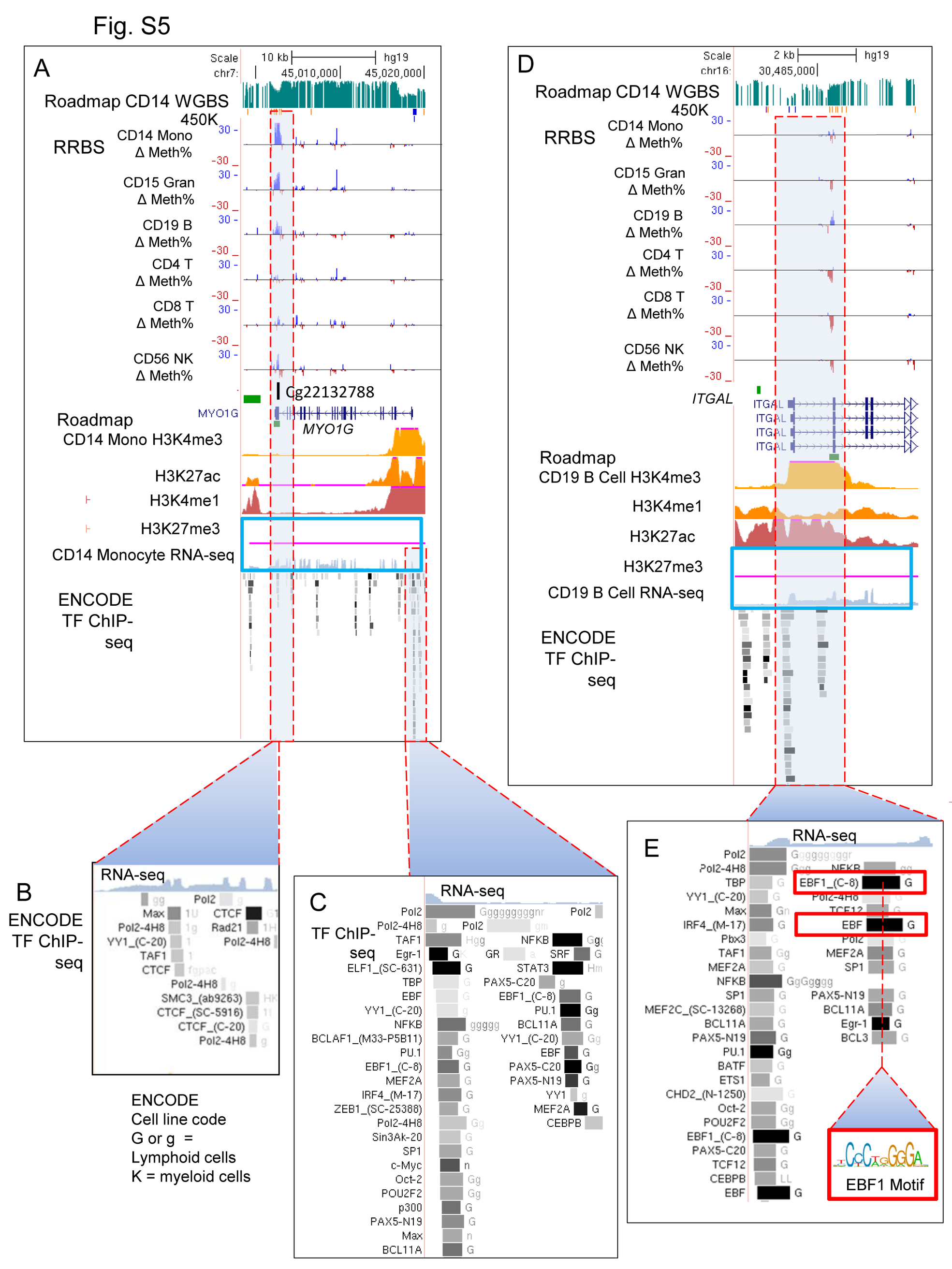

Supplement: S5 Fig — (A) Increased levels of CpG methylation were observed in MYO1G across a ~500-bp gene regulatory region (B) with greatest increase in methylation in CD14+ monocytes. (C) The promoter of the highly expressed MYO1G gene displays activating histone marks and transcription factor binding. (D) ITGAL, sometimes called CD11A, shows a unique DMR in CD19+ B cells. It displays activating histone modifications and is highly expressed in CD19+ B cells. (E) EBF1, a B cell specific TF occupies its binding motif near the DMR in B lymphoblastoid cells. (TIF) [file pone.0166486.s005.tif]

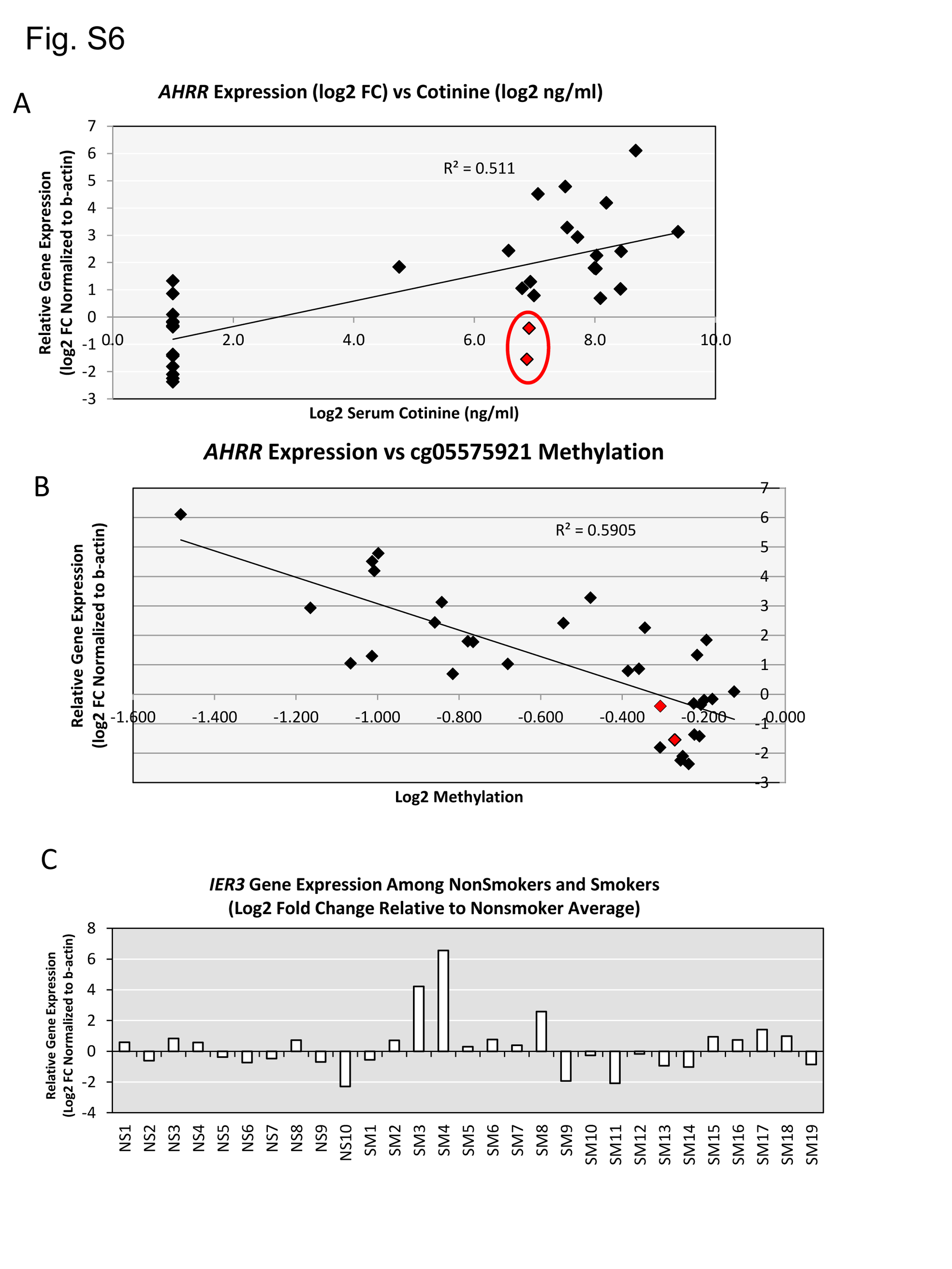

Supplement: S6 Fig — (A) AHRR mRNA expression (RT-PCR, log2 fold change relative to nonsmokers) in monocytes vs serum cotinine values. Cotinine outliers marked by red. (B) AHRR mRNA levels (log2 fold change) in monocytes vs methylation (log2) in monocytes. Cotinine outliers (red) were not outliers for methylation vs expression, suggestive of a secondary exposure to nicotine such as eCigarettes. (C) Individual IER3 log2 fold change expression values in B cells for nonsmokers (n = 10) and smokers (n = 19) relative to nonsmoker mean. (TIF) [file pone.0166486.s006.tif]
