# Supplementary material for: Distinct Epigenetic Effects of Tobacco Smoking in Whole Blood and among Leukocyte Subtypes
Source: PLoS One. 2016 Dec 9;11(12):e0166486. doi: 10.1371/journal.pone.0166486 (PMC5147832; doi:10.1371/journal.pone.0166486)
Supplement: S1 File — (DOCX) [file pone.0166486.s007.docx]

**Supporting Information Methods**

**Distinct Epigenetic Effects of Tobacco Smoking in Whole Blood and Leukocyte Subtypes**

Dan Su, Xuting Wang, Michelle R. Campbell, Devin K. Porter, Gary S. Pittman, Brian D. Bennett, Ma Wan, Neal Englert, Christopher L. Crowl, Ryan N. Gimple, Kelly N. Adamski, Zhiqing Huang, Susan K. Murphy, and Douglas A. Bell

**Study populations.** 253 individual study participants consisting of 172 smokers and 81 non-smokers enrolled between 1993 and 1995 as healthy volunteers from the general public in Durham and Chapel Hill, North Carolina. These subjects were part of a community-based sample comprised of 294 healthy unrelated blacks and whites and have been described in several studies as disease-free control smokers or non-smokers ([Jones et al. 1993](#_ENREF_4); [Bell et al. 1995](#_ENREF_5); [Li et al. 2000](#_ENREF_32); [Lunn et al. 1999](#_ENREF_33)).

Nucleated DNA from whole blood was collected after sucrose-induced osmotic lysis of cells followed by phenol-chloroform extraction and DNA was stored in Tris-EDTA buffer at -20°C. At collection time, subjects were aged 19-63 years old. Selection of subjects was based upon available DNA sample; self-reporting of no recent illness; no prior exposure to radiation or chemicals other than smoking or nonprescription medications; no prior history of heart disease, diabetes, tuberculosis, high blood pressure, cancer, or hepatitis; no passive cigarette/cigar/marijuana smoke exposure at home; all non-smokers were self-reported as not having smoked >100 cigarettes in their lifetime. Smokers reported their average daily cigarette consumption for the past 3 months. Age and smoking history of all subjects are given in Table S1 as averages and range. The analysis of samples was carried out under an approved human subject protocol (NIEHS 86-E-0037).

An independent study of females (20 smokers and 14 non-smokers) of African-American or European-American was recruited at the NIEHS Clinical Research Unit (protocol 10-E-0063) between March 2013 and January 2015 from the Raleigh, Durham and Chapel Hill, NC area. Selection of subjects was also based upon self-reporting of no illness or diseases using similar criteria as listed above. Peripheral blood monocytes, lymphocytes, eosinophils, basophils, and neutrophils were counted by an automated cell counter, Coulter HmX AL Hematology Analyzer (Beckman Coulter, UK) by the Hematological Laboratory at NIEHS. Average age, smoking history, and WBC components of participants are given in Table S2. For all participants, serum was taken for nicotine/cotinine levels measured by HPLC-MS, with undetectable levels of cotinine reported as < 2 ng/mL. Smoking status was self-reported and confirmed by cotinine levels: 13 non-smokers with undetectable cotinine and one with 27 ng/mL, and 20 smokers with cotinine ranging from 38 – 666 ng/mL.

**Peripheral blood leukocyte subtype isolation.** Whole blood was fractionated by density gradient centrifugation using Histopaque-1077 Ficoll medium and Accuspin™ Tubes (Sigma-Aldrich). Mononuclear cell fractions were extracted from the serum/medium boundary. Granulocytes were isolated directly from whole blood using anti-CD15+ antibody–coated magnetic beads (Invitrogen). The mononuclear layer was used for isolation of CD14+ monocytes, CD2+ T lymphocytes and CD19+ B lymphocytes using antibody-coated magnetic beads (Invitrogen). Blood samples were processed within 4 hours after blood draw. Based on post hoc assessment of cell type using cell-type specific methylation signature developed by Houseman et al. ([2012](#_ENREF_28)), and based on the cell type reference data from Reinius et al. ([2012](#_ENREF_27)) (see Figure S2 and Table S2), all samples of the CD14+ monocyte fraction were consistently 95-100% pure with an average purity of 99%. Purity of all samples for CD15+ granulocyte fraction, CD2+ cell fraction, and CD19+ B cell fraction was between 92-100%, 97-100%, and 90-100%, respectively. However, PBMCs contained small amounts of granulocytes (~5%). DNA from purified, leukocyte subtypes was extracted using QIAGEN All Prep kit (QIAGEN).

**Infinium human methylation assays.** The Human Methylation 450 BeadChip (Illumina) was used to measure methylation by the NCI Center for Genome Research. Specifically, 500 ng of DNA was bisulfite converted using the EZ-DNA Methylation kit (Zymo Research), hybridized to HumanMethylation450 BeadChip arrays and then scanned with an iScan microarray scanner (Illumina) following the manufacturer’s protocols. The ChAMP pipeline was used to extract and analyze data from iDat files ([Morris et al. 2014](#_ENREF_34)). Probes with SNPs (MAF >= 0.01 in 1000 Genomes Database) present at target sites were excluded.

**Statistical analyses.** To investigate the association between current smoking and DNA methylation, normalized and batch-corrected beta-values were transformed to log ratio, defined as log_2_[β/(1 – β)], and then fitted using robust linear regression ([Fox and Weisberg 2011](#_ENREF_37)) adjusted for age, sex, race and cell type counts. Cell types ("CD4T", "CD8T", "Bcell", "Mono", "NK", "Neu", "Eos") and their counts in whole blood were estimated using the method of Houseman et al. ([2012](#_ENREF_28)) . The regression results were further corrected for multiple testing at a false discovery rate (FDR) of 0.05 using the method of Benjamini and Hochberg ([1995](#_ENREF_38)). To explore the associated between years of smoking in heavy smokers, we considering the top 1000 CpGs associated with any level of smoking, then we carried out a stratified analysis (above and below median, <22 cigarettes/day versus >22 cigarettes/day) and ranked CpGs by p-value for association with years of smoking in each stratum. We then calculated the difference in rank across the strata for each CpG.

**Enrichment analysis of methylation regions associated with smoking.**  We used the GREAT (Genomic Regions Enrichment of Annotations Tool, <http://bejerano.stanford.edu/great/public/html/>) (McLean et al. 2010) to find enriched functional terms of genes near our top 738 CpGs as these terms indicate the potential regulatory functions of these CpGs.  Each CpG probe was first assigned a gene regulatory domain that extends in both directions to the midpoint between the gene's TSS and the nearest gene's TSS but no more than 100kb in one direction; then the GREAT was run with default parameters. We focused on the enrichments have the following properties: (1) regions hits > 100; (2) enrichment fold > 2; and (3) FDR q < 0.05. If a term is a parent of another term based on the ontology tree, then the parent term is removed. All enrichment tests were calculated by Fisher’s Exact test, and differences in methylation level between groups was calculated by t-test.

**Reduced Representation Bisulfite Sequencing.** To investigate the relationship between smoking and CpG methylation not captured by microarray, and to qualitatively visualize DMRs, RRBS libraries were constructed (as in reference Ziller et al and Reynolds et al) from DNA extracted from CD14+ monocytes, CD15+ granulocytes, CD19+ B cells, CD4+ T cells, CD8+, and CD56 NK cells from 5 smokers and 5 nonsmokers and sequenced on Illumina HiSeq 2500 at the NIH Intramural Sequencing Center. Briefly, 200ng genomic DNA was spike-in with 50pg phage lambda DNA for bisulfite conversion control. Samples were digested with Msp I (C^CGG, New England Biolabs) overnight and purified by 2x volume of AMPure XP beads (Beckman Coulter) followed by end repairing, A-tailing, and indexed adapter ligation using TruSeq DNA Sample Preparation Kit v2 (Illumina). Ligated DNA samples were purified with 2x volume of AMPure XP beads followed by two consecutive bisulfite conversions using EpiTect Bisulfite kit. Bisulfite converted DNA samples were purified with 2.5x volume of AMPure XP beads before 9 cycles of PCR amplification for library generation and cleaned up with 1.2x volume of AMPure XP beads clean-up. To further minimize adapter dimers, a second round of clean-up was conducted with 1.5x volume of AMPure XP beads. The final library DNA samples were eluted with 32.5 ul resuspension buffer from TruSeq DNA Sample Preparation Kit. Libraries were quantified by a Qubit fluorometer (Invitrogen). The size distribution and concentration of DNA fragments was tested on a High Sensitivity DNA Chip (Agilent Technologies) on Agilent 2100 Bioanalyzer. Ten-plex RRBS libraries were pooled and run on the HiSeq 2500.

**RRBS-seq Processing.** We filtered raw sequence reads to only include those with a median Phred quality score of 20 or greater. We trimmed reads containing adapters (Trim Galore! version 0.2.8) the first two bases of every mate 2 read and the last two bases of any mate 1 read that had been trimmed of adapter sequence. We aligned the filtered and trimmed reads to the hg19 genome assembly using Bismark version 0.9.0. To avoid double counting bases that originated from the same fragment but were present in both paired end reads, we trimmed all bases from any mate 2 read that overlapped with its paired mate 1 read. We extracted the methylation status of all CpGs within the reads, and we assembled a data set with the counts of methylated and unmethylated cytosines for each CpG and sample. To avoid spurious results due to SNP differences being quantified as methylation differences, we eliminated any CpGs that were located at known common SNPs, which were defined as being present in dbSNP build 138 at a population frequency of 1% or greater. To visualize smoking-associated differentially methylated regions, the average methylation level at each captured CpG for 5 nonsmokers was subtracted from the average methylation level for 5 smokers. The changes in methylation percent for each cell type were plotted at each RRBS captured CpG (Figure 6, S4, S5) and increases in methylation are plotted as positive bars (blue) while decreases are plotted as negative bars (red).

**Reverse transcriptase quantitative polymerase chain reaction (RT-qPCR).** Gene expression levels of AHRR, GPR15, F2RL3, ALPPL2, IER3 and GFI1 were determined in mRNA extracted from cell types isolated with antibody coated beads from blood as described above. RNA and DNA were isolated using the ALLPrep DNA/RNA/miRNA Universal Kit (Qiagen). cDNA was generated using the SuperScript® III First-Strand Synthesis (Life Technologies). For each individual RNA sample, target and reference genes (*AHRR, GPR15, F2RL3, IER3*, *GFI1, ITGAL* and ß-actin) were amplified in triplicate using TaqMan assays (Life Technologies AHRR: Hs01005075_m1; GPR15: Hs00922903_s1; β-actin: Hs01060665_g1; F2RL3: Hs01006385_g1; ALPPL2: Hs00741068_g1; IER3: Hs04187506_g1; GFI1: Hs00382207_m1) designed to span exon junctions using Universal PCR Master Mix (Life Technologies) and the ABI 7900HT Real-time PCR machine. Gene level data from individual samples was normalized to b-actin and fold change differences were assessed relative to non-smokers using the delta-delta Ct method. Poor quality RNA samples or low yield samples were omitted from analysis, specifically CD15+ granulocytes produced quality RNA for only 9 nonsmokers, and 11 smokers. Gene expression levels for AHRR in CD14+ monocytes for 10 individual subjects were previously reported in Reynolds et al. 2015.

**Supporting Information References**

Bell, D.A., Liu, Y., and Cortopassi, G.A. (1995). Occurrence of bcl-2 oncogene translocation with increased frequency in the peripheral blood of heavy smokers. J Natl Cancer Inst *87*, 223-224.

Benjamini, Y. and Hochberg,Y. ([1995](#_ENREF_38)). Controlling the false discovery rate: a practical and powerful approach to multiple testing. J R Stat Soc Series B Stat Methodol *57,* 289-300.

Fox, J., and Weisberg, S. (2011). An R Companion to Applied Regression. 449.

Houseman, E.A., Accomando, W.P., Koestler, D.C., Christensen, B.C., Marsit, C.J., Nelson, H.H., Wiencke, J.K., and Kelsey, K.T. (2012). DNA methylation arrays as surrogate measures of cell mixture distribution. BMC bioinformatics *13*, 86.

Jones, I.M., Moore, D.H., Thomas, C.B., Thompson, C.L., Strout, C.L., and Burkhart-Schultz, K. (1993). Factors affecting HPRT mutant frequency in T-lymphocytes of smokers and non-smokers. Cancer epidemiology, biomarkers & prevention: *2*, 249-260.

Li, R.L., Boerwinkle, E., Olshan, A.F., Chambless, L.E., Pankow, J.S., Tyroler, H.A., Bray, M., Pittman, G.S., Bell, D.A., and Heiss, G. (2000). Glutathione S-transferase genotype as a susceptibility factor in smoking-related coronary heart disease. Atherosclerosis *149*, 451-462.

Lunn, R.M., Langlois, R.G., Hsieh, L.L., Thompson, C.L., and Bell, D.A. (1999). XRCC1 polymorphisms: effects on aflatoxin B1-DNA adducts and glycophorin A variant frequency. Cancer Res *59*, 2557-2561.

Mao, B., Zhang, Z., and Wang, G. (2015). BTG2: a rising star of tumor suppressors (review). International Journal of Oncology *46*, 459-464.

Markunas, C.A., Xu, Z., Harlid, S., Wade, P.A., Lie, R.T., Taylor, J.A., and Wilcox, A.J. (2014). Identification of DNA methylation changes in newborns related to maternal smoking during pregnancy. Environ Health Perspect. *122*(10), 1147-53.

McLean, C.Y., Bristor, D., Hiller, M., Clarke, S.L., Schaar, B.T., Lowe, C.B., Wenger, A.M., and Bejerano, G. (2010). GREAT improves functional interpretation of cis-regulatory regions. Nat Biotechnol. *28*(5), 495-501.

Morris, T.J., Butcher, L.M., Feber, A., Teschendorff, A.E., Chakravarthy, A.R., Wojdacz, T.K., and Beck, S. (2014). ChAMP: 450k Chip Analysis Methylation Pipeline. Bioinformatics *30*, 428-430.

Parr, C., and Jiang, W.G. (2009). Metastasis suppressor 1 (MTSS1) demonstrates prognostic value and anti-metastatic properties in breast cancer. Eur J Cancer *45*, 1673-1683.

Reinius, L.E., Acevedo, N., Joerink, M., Pershagen, G., Dahlen, S.E., Greco, D., Soderhall, C., Scheynius, A., and Kere, J. (2012). Differential DNA methylation in purified human blood cells: implications for cell lineage and studies on disease susceptibility. PLoS One *7*, e41361.

Reynolds, L., Wan, M., Ding, J., Taylor, J.R., Lohman, K.K., Su, D., Bennett, B., Porter, D., Gimple, R., Pittman, G.S.*, et al.* (2015). DNA Methylation of the Aryl Hydrocarbon Receptor Repressor Associations with Cigarette Smoking and Subclinical Atherosclerosis. Circulation: Cardiovascular Genetics  *8*, 707-716.
