# Supplementary material for: Distinct Epigenetic Effects of Tobacco Smoking in Whole Blood and among Leukocyte Subtypes
Source: PLoS One. 2016 Dec 9;11(12):e0166486. doi: 10.1371/journal.pone.0166486 (PMC5147832; doi:10.1371/journal.pone.0166486)
Supplement: S1 Table — (DOCX) [file pone.0166486.s008.docx]

**Table S1**. Smoking associated CpG sites (ever/never, p<1.2 x 10^-7^), pack-years, previous report.

Abbreviations: Chr: chromosome, coef: coefficient, Rsq: R-square, Rsq-Adj: adjusted R-square, NA: not applicable

**^a^**Association with smoking after adjustment for age, sex, race and cell-type counts and reach genome-wide significance (Bonferroni-corrected threshold 1.2E-07) (p-value, coefficient). Corresponding results of the same CpG sites for pack-year association after adjustment for age, sex, race (p-value). **^b^**Corresponding results of the same CpG sites for pack-year association after adjustment for age, sex, race and cell-type counts (p-value, Q value, coefficient, adjusted R-square). The data sorted by the p-value for association with smoking after cell-type count correction are organized by the chromosome and mapinfo (Genome build 37). **^c^**"Yes" denotes significant dose-response (pack-year association) after adjustment for age, sex, race and cell -type counts and reach genome-wide significance (Bonferroni-corrected threshold 1.2E-07). **^d^**Reports of the replication of the site using previously published studies that utilized Illumina HumanMethylation450 BeadChips. Study abbreviations: Z: Zeilinger, S et al. 2013 F4 discovery +F3 replication panel, Z*: Zeilinger, S et al. 2013 F4 discovery but not get significant in F3 replication panel, D: Dogan,MV 2014, E: Elliott, HR et al. 2014,T: Tsaprouni LG et al. 2014, J: Joubert, BR et al. 2012, and M: Markunas,CA et al. 2014. X^a^ According to UCSC Genome Browser no annotated transcripts are associated with these CpG sites.
