# Supplementary material for: Distinct Epigenetic Effects of Tobacco Smoking in Whole Blood and among Leukocyte Subtypes
Source: PLoS One. 2016 Dec 9;11(12):e0166486. doi: 10.1371/journal.pone.0166486 (PMC5147832; doi:10.1371/journal.pone.0166486)
Supplement: S2 Table — (DOCX) [file pone.0166486.s009.docx]

**Supporting Information Table S2.** GREAT Functional Analysis

| **Category** | **Ontology** | **p-Value** | **FDR** | **Enrichment** | **Region Hits** | **Gene Hits** | **Total Genes** | **Gene Set Coverage** |
| --- | --- | --- | --- | --- | --- | --- | --- | --- |
| Disease Ontology | hematologic cancer | 1.28E-24 | 5.72E-22 | 3.09 | 107 | 69 | 1422 | 0.135 |
| Disease Ontology | hematopoietic system disease | 7.61E-23 | 2.43E-20 | 2.85 | 111 | 73 | 1631 | 0.142 |
| Disease Ontology | nervous system cancer | 1.83E-18 | 3.41E-16 | 2.64 | 101 | 69 | 1480 | 0.135 |
| Disease Ontology | cardiovascular system disease | 1.66E-23 | 6.17E-21 | 2.43 | 146 | 103 | 2507 | 0.201 |
| Disease Ontology | glandular and epithelial neoplasm | 1.13E-18 | 2.30E-16 | 2.39 | 120 | 88 | 2013 | 0.172 |
| Mammalian Phenotype | abnormal cell differentiation | 2.96E-29 | 7.81E-26 | 3.33 | 115 | 76 | 1283 | 0.148 |
| Mammalian Phenotype | abnormal mononuclear cell morphology | 3.51E-23 | 1.46E-20 | 3.04 | 103 | 70 | 1448 | 0.136 |
| Mammalian Phenotype | abnormal skeleton morphology | 3.09E-24 | 1.75E-21 | 2.99 | 110 | 88 | 1357 | 0.172 |
| Mammalian Phenotype | abnormal hematopoietic system morphology/development | 1.99E-24 | 1.31E-21 | 2.64 | 133 | 96 | 2184 | 0.187 |
| Mammalian Phenotype | preweaning lethality | 7.51E-27 | 6.61E-24 | 2.26 | 188 | 148 | 3259 | 0.288 |
| Promoter Motif | Motif GGGAGGRR matches MAZ binding site | 4.07E-21 | 2.50E-18 | 2.50 | 126 | 96 | 2181 | 0.187 |
| Promoter Motif | Motif TGGAAA matches NFAT binding site | 6.72E-13 | 4.13E-11 | 2.12 | 104 | 78 | 1803 | 0.152 |
| Promoter Motif | Motif CAGGTG matches TCF3 binding site | 8.71E-15 | 1.07E-12 | 2.08 | 124 | 98 | 2371 | 0.191 |
| GO: Molecular Function | DNA binding | 7.11E-35 | 3.75E-32 | 3.00 | 156 | 111 | 2381 | 0.216 |
| GO: Biological Process | RNA biosynthetic process | 4.13E-42 | 3.08E-39 | 3.16 | 174 | 122 | 2506 | 0.238 |
| GO: Biological Process | gene expression | 3.46E-39 | 1.51E-36 | 2.73 | 197 | 142 | 3431 | 0.277 |
| GO: Biological Process | regulation of biosynthetic process | 3.61E-40 | 1.88E-37 | 2.55 | 221 | 162 | 3763 | 0.316 |
| GO: Biological Process | regulation of nitrogen compound metabolic process | 4.17E-42 | 2.90E-39 | 2.52 | 235 | 172 | 4015 | 0.335 |
| GO: Biological Process | anatomical structure development | 1.34E-39 | 6.34E-37 | 2.40 | 239 | 186 | 3888 | 0.363 |
| GO: Biological Process | multicellular organismal development | 1.46E-39 | 6.64E-37 | 2.37 | 243 | 194 | 3973 | 0.378 |
