# Supplementary material for: Distinct Epigenetic Effects of Tobacco Smoking in Whole Blood and among Leukocyte Subtypes
Source: PLoS One. 2016 Dec 9;11(12):e0166486. doi: 10.1371/journal.pone.0166486 (PMC5147832; doi:10.1371/journal.pone.0166486)
Supplement: S3 Table — (DOCX) [file pone.0166486.s010.docx]

| **Probe**  **Table S3.** GREAT Enrichment Hematological Cancers | Gene (distance to TSS) | **Ontological Pathway** |
| --- | --- | --- |
| **cg27005749** | AGO2 (+37071) | **hematologic cancer** |
| **cg26291848** | AGO2 (+37401) | **hematologic cancer** |
| **cg23161492** | ANPEP (+891) | **hematologic cancer** |
| **cg25707298** | BNIP3 (-3986) | **hematologic cancer** |
| **cg01460227** | CALCR (-45) | **hematologic cancer** |
| **cg08402433** | CARD11 (+63423) | **hematologic cancer** |
| **cg24613080** | CCL2 (-98268) | **hematologic cancer** |
| **cg19942495** | CCL2 (-98276) | **hematologic cancer** |
| **cg26286082** | CCNK (+56646) | **hematologic cancer** |
| **cg11258381** | CD2 (+53688) | **hematologic cancer** |
| **cg23090529** | CDKN2C (+15717) | **hematologic cancer** |
| **cg14781374** | CHUK (-9030) | **hematologic cancer** |
| **cg04945379** | CIITA (-854) | **hematologic cancer** |
| **cg03544320** | CRMP1 (+93) | **hematologic cancer** |
| **cg07632771** | EZH2 (-30454) | **hematologic cancer** |
| **cg00489401** | FLT4 (+748) | **hematologic cancer** |
| **cg09935388** | GFI1 (+1922) | **hematologic cancer** |
| **cg18316974** | GFI1 (+2475) | **hematologic cancer** |
| **cg12876356** | GFI1 (+2685) | **hematologic cancer** |
| **cg18146737** | GFI1 (+2810) | **hematologic cancer** |
| **cg09662411** | GFI1 (+3378) | **hematologic cancer** |
| **cg10399789** | GFI1 (+3842) | **hematologic cancer** |
| **cg00383220** | H2AFX (+2296) | **hematologic cancer** |
| **cg19293468** | HIC1 (+13797) | **hematologic cancer** |
| **cg24173182** | HIC1 (+1683) | **hematologic cancer** |
| **cg17350632** | HLA-DPB1 (+2033) | **hematologic cancer** |
| **cg19513004** | HMGA1 (+2034) | **hematologic cancer** |
| **cg17131837** | HMGA2 (+33751) | **hematologic cancer** |
| **cg14826425** | HOXA13 (+6887) | **hematologic cancer** |
| **cg18311537** | HOXA9 (-4047) | **hematologic cancer** |
| **cg04010091** | HOXB9 (-10331) | **hematologic cancer** |
| **cg11546137** | HOXD11 (+550) | **hematologic cancer** |
| **cg07536910** | HOXD11 (-23908), HOXD13 (-9513) | **hematologic cancer** |
| **cg03964958** | HOXD11 (-7293) | **hematologic cancer** |
| **cg06126421** | IER3 (-7750) | **hematologic cancer** |
| **cg14753356** | IER3 (-7778) | **hematologic cancer** |
| **cg24859433** | IER3 (-7873) | **hematologic cancer** |
| **cg15342087** | IER3 (-7879) | **hematologic cancer** |
| **cg26316423** | IL2RA (+150) | **hematologic cancer** |
| **cg16462648** | IL6ST (-94430) | **hematologic cancer** |
| **cg09099830** | ITGAL (+1507) | **hematologic cancer** |
| **cg16391678** | ITGAL (+1619) | **hematologic cancer** |
| **cg06235438** | ITGAL (+1988) | **hematologic cancer** |
| **Probe** | Gene (distance to TSS) | **Ontological Pathway** |
| **cg06972908** | ITGAL (+4343) | **hematologic cancer** |
| **cg14316231** | KAT6A (+14404) | **hematologic cancer** |
| **cg04299274** | KSR1 (-294) | **hematologic cancer** |
| **cg24147838** | LAIR1 (-101) | **hematologic cancer** |
| **cg23162201** | LMO1 (+4663) | **hematologic cancer** |
| **cg21611682** | LRP5 (+58193) | **hematologic cancer** |
| **cg22950163** | MMP2 (+643) | **hematologic cancer** |
| **cg08269389** | MR1 (+70871) | **hematologic cancer** |
| **cg24142603** | MSC (+2814) | **hematologic cancer** |
| **cg26427498** | NAMPT (-61621) | **hematologic cancer** |
| **cg04347477** | NCOR2 (-22210) | **hematologic cancer** |
| **cg04704064** | NCOR2 (-59422) | **hematologic cancer** |
| **cg13015710** | NCOR2 (-59546) | **hematologic cancer** |
| **cg25716814** | NFIX (+18539) | **hematologic cancer** |
| **cg11183632** | NKX2-2 (-8489) | **hematologic cancer** |
| **cg14406134** | ORM1 (-70413) | **hematologic cancer** |
| **cg02606423** | OTX1 (+2440) | **hematologic cancer** |
| **cg15473329** | PAF1 (+18916) | **hematologic cancer** |
| **cg26669717** | PDGFA (-81998) | **hematologic cancer** |
| **cg21381845** | PDGFA (-83097) | **hematologic cancer** |
| **cg03226435** | PRDM16 (+72425) | **hematologic cancer** |
| **cg19243842** | PRDM16 (+72747) | **hematologic cancer** |
| **cg10111335** | PREP (-84957) | **hematologic cancer** |
| **cg18395636** | RAB38 (-151) | **hematologic cancer** |
| **cg04086928** | RAPGEF1 (-27416) | **hematologic cancer** |
| **cg07285276** | RAPGEF1 (-27787) | **hematologic cancer** |
| **cg19572487** | RARA (+10579) | **hematologic cancer** |
| **cg13940444** | RARG (+8653) | **hematologic cancer** |
| **cg05656688** | RUNX3 (+37412) | **hematologic cancer** |
| **cg15963463** | RUNX3 (+38263) | **hematologic cancer** |
| **cg08544331** | RUNX3 (+44578) | **hematologic cancer** |
| **cg15498134** | RUNX3 (+44646) | **hematologic cancer** |
| **cg00147638** | RUNX3 (+63461) | **hematologic cancer** |
| **cg12459932** | RUNX3 (-518) | **hematologic cancer** |
| **cg23916689** | RUNX3 (-534) | **hematologic cancer** |
| **cg27537125** | RUNX3 (-58181) | **hematologic cancer** |
| **cg19774846** | RUNX3 (-725) | **hematologic cancer** |
| **cg13898430** | RUNX3 (-774) | **hematologic cancer** |
| **cg16220183** | SEPT4 (+8491) | **hematologic cancer** |
| **cg05115106** | SEPT9 (-27147) | **hematologic cancer** |
| **cg01979157** | SKI (+880) | **hematologic cancer** |
| **cg24587080** | SOD2 (+97986) | **hematologic cancer** |
| **cg16794961** | SOX11 (+49234) | **hematologic cancer** |
| **cg07065111** | SOX11 (-1585) | **hematologic cancer** |
| **Probe** | Gene (distance to TSS) | **Ontological Pathway** |
| **cg01681367** | SPN (+1492) | **hematologic cancer** |
| **cg10089801** | SUMO1 (+67095) | **hematologic cancer** |
| **cg09287629** | SUMO1 (+67122) | **hematologic cancer** |
| **cg08621447** | SUMO1 (+67183) | **hematologic cancer** |
| **cg23340017** | TLX1 (+5877) | **hematologic cancer** |
| **cg21393163** | TNFRSF1B (-9430) | **hematologic cancer** |
| **cg24427243** | TNFRSF8 (+4667) | **hematologic cancer** |
| **cg20462100** | TNFRSF8 (-18562) | **hematologic cancer** |
| **cg07094298** | TNIP2 (+10076) | **hematologic cancer** |
| **cg00741986** | TNIP2 (+9770) | **hematologic cancer** |
| **cg06939447** | TP73 (+20888) | **hematologic cancer** |
| **cg22383924** | TP73 (+21396) | **hematologic cancer** |
| **cg14368473** | TRAF1 (-3671) | **hematologic cancer** |
| **cg15107670** | WT1 (+416) | **hematologic cancer** |
| **cg18484958** | WWTR1 (+74) | **hematologic cancer** |
| **cg10827488** | ZBTB16 (+23524) | **hematologic cancer** |
| **cg18295744** | ZMIZ1 (+44135) | **hematologic cancer** |
| **cg03450842** | ZMIZ1 (+6156) | **hematologic cancer** |
