# Supplementary material for: Distinct Epigenetic Effects of Tobacco Smoking in Whole Blood and among Leukocyte Subtypes
Source: PLoS One. 2016 Dec 9;11(12):e0166486. doi: 10.1371/journal.pone.0166486 (PMC5147832; doi:10.1371/journal.pone.0166486)
Supplement: S4 Table — (DOCX) [file pone.0166486.s011.docx]

Table S4. CpGs associated with years of smoking among heavy smokers (>22 cigarettes per day), but not light smokers.
